# Psoralidin, a main compound in Psoraleae Fructus, induces hepatotoxicity by impeding lipid oxidative catabolism and aggravating lipid accumulation in mice

**DOI:** 10.1186/s13020-026-01335-x

**Published:** 2026-02-02

**Authors:** Zhaojuan Guo, Xiyi Peng, Dasheng Qin, Lin Zhang, Can Tu, Ting Wang

**Affiliations:** 1 Beijing Research Institute of Chinese Medicine, Beijing University of Chinese Medicine, Yangguang South Street, Fang-Shan District, Beijing, 102488 China; 2 NMPA Center for Innovation and Research in Regulatory Science , Beijing University of Chinese Medicine, Beijing, 102488 China; 3 Present Address: Beijing International Science and Technology Cooperation Base for TCM Hepatotoxicity and New Drug Research and Development, Beijing University of Chinese Medicine, Beijing, 102488 China

**Keywords:** Hepatotoxicity, Psoraleae Fructus, Psoralidin, Lipid synthesis, Lipid metabolism, Multi-omics

## Abstract

**Background:**

*Psoralea corylifolia*(PF) is widely utilized for the treatment of conditions such as kidney yang deficiency, frequent urination, and cold pain in the waist and knees. However, both basic research and clinical reports indicate that it induce hepatotoxicity. Our preliminary research has confirmed that PF has hepatotoxicity and in vitro research indicated that psoralidin is hepatotoxic. but it remains unclear whether psoralidin is the hepatotoxic component of PF and the mechanism of psoralidin induces hepatotoxicity. This study aimed to investigate the hepatotoxicity induced by psoralidin and its toxic mechanisms.

**Methods:**

Kunming mice were used to conduct long-term toxicity experiments. Liver function indices, organ coefficients, and histopathological observations were employed to assess the hepatotoxicity of psoralidin. Non-targeted metabolomics and proteomics analyses were conducted to elucidate the potential pathways and targets associated with psoralidin-induced hepatotoxicity. Furthermore, immunofluorescence staining, molecular docking and Western blotting analyses were utilized to validate the mechanisms underlying psoralidin hepatotoxicity.

**Results:**

The elevation of ALT and AST, accompanied by hepatic steatosis and lipid droplet aggregation were observed after psoralidin treatement. Psoralidin affected biosynthesis of unsaturated fatty acid, fatty acid metabolism, arachidonic acid metabolism, phospholipid metabolism, and oxidative phosphorylation. Further validation research found that psoralidin induced the expressions of Acot4 and Plin5, which in turn caused up-regulations of TGs and FFA in mice, and increased the HSD17B12 level, thereby promoting the synthesis of long-chain fatty acids and facilitating lipid synthesis. And psoralidin catalyzed the conversion of phosphatidylcholine into LPC by enhancing Pla2g6 and Pla2g12b levels, which promoted the synthesis and accumulation of TGs, ultimately inducing disorders in glycerophospholipid metabolism. Furthermore, psoralidin caused upregulation of ROS and mitochondrial damage, leading to a decrease in FA oxidation.

**Conclusion:**

Psoralidin is one of the hepatotoxic components of PF, which induced hepatotoxicity via promoting lipid synthesis and inhibiting lipid oxidative degradation.

**Graphical Abstract:**

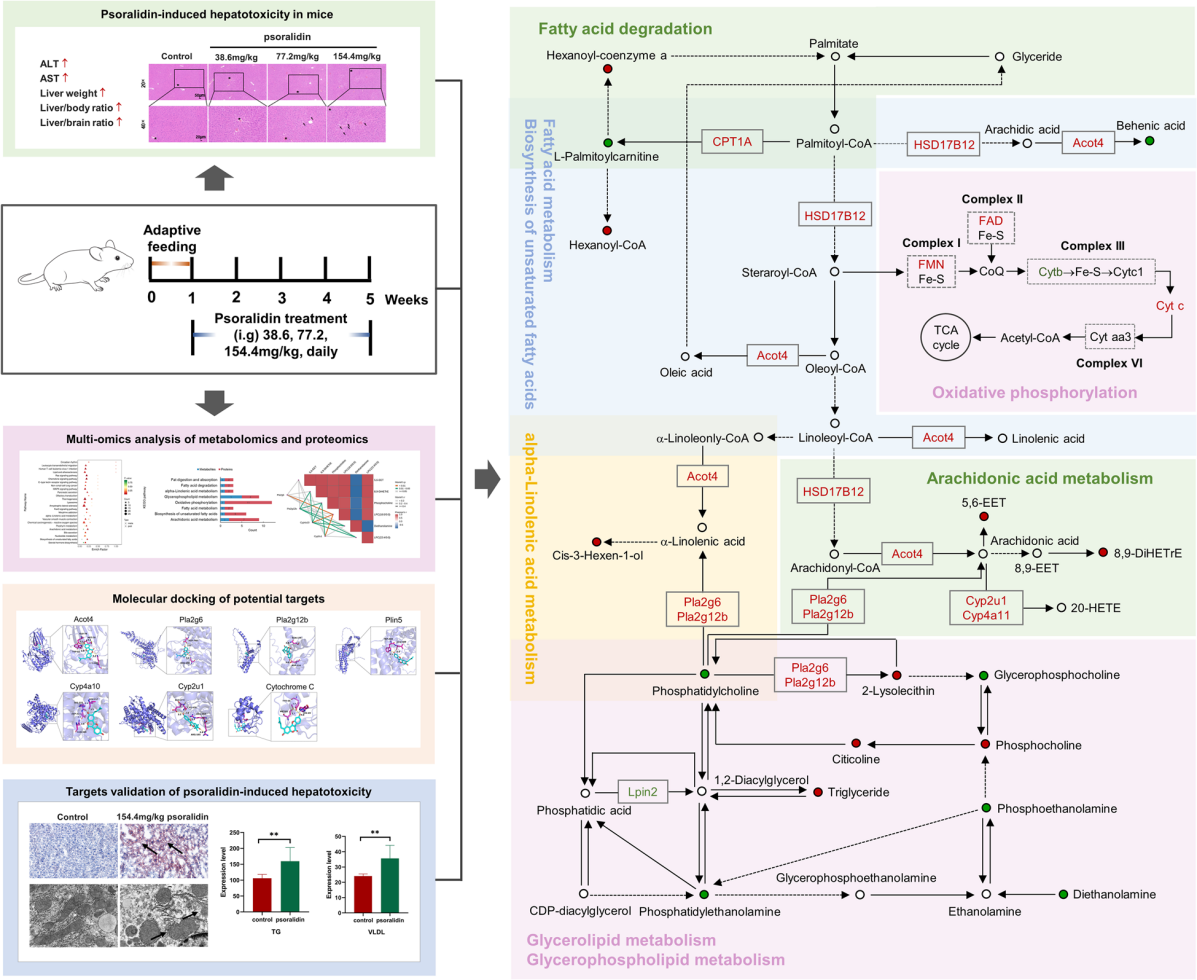

**Supplementary Information:**

The online version contains supplementary material available at 10.1186/s13020-026-01335-x.

## Introduction

In recent years, the widespread application of traditional Chinese medicine (TCM) has been accompanied by frequent reports of safety concerns. Liver injury is one of the most common adverse effects of herbs and dietary supplements (HDS), and HDS-induced liver injury accounts for 20% of hepatotoxicity cases in the United States. In China, 26.81% of liver injuries are caused by herbal medicines and dietary supplements [1, 2]. LiverTox, a free database of liver injury-related medications in the United States, includes more than 30 herbs that can cause drug‑induced liver injury (DILI), such as *Psoraleae Fructus* (PF) and *Polygoni Multiflori Radix* (PMR). Herb-induced liver injury (HILI) has emerged as a prevalent adverse reaction in clinical settings, particularly with traditionally regarded "non-toxic" Chinese medicines, such as PF, *Dictamni Cortex*, and PMR, which have been associated with severe adverse reactions, including acute liver failure and death, thus raising significant concerns about the safety of TCM. In 2024, TCM-related adverse reactions accounted for 12.1% of the total adverse drug reaction/event reports on average of our country [3]. The toxicity of TCM has been recognized as a critical issue in the "Major Scientific Issues and Engineering Technical Problems of Chinese Medicine in 2021" [4–7].

PF, derived from the dried and mature fruit of the leguminous plant *Psoralea corylifolia* L., is widely utilized in clinical practice for the treatment of conditions such as kidney yang deficiency, frequent urination, and cold pain in the lower back and knees [8]. As an traditional Chinese herbal medicine, PF has a long history of clinical application in Asia, especially in China, Japan, and South Korea. It is widely used in the treatment of osteoporosis, fractures, vitiligo and other diseases [9, 10]. It is worth noting that ancient books of herb medicine have not recorded liver toxicity associated with PF. However, in recent years, adverse reactions related to PF and Chinese patent medicine containing PF have occurred frequently. Reports from the National Adverse Drug Reaction Monitoring Center have documented cases of liver injury linked to Chinese patent medicines containing PF, leading to product label revisions to include liver damage as a potential adverse reaction. Instances of abnormal biochemical markers and severe liver injury have been reported [11–13]. In addition, there have been reported cases of liver injury caused by PF-related preparations in countries such as China, South Korea, Germany, and India.[10, 14–16]. In our previous safety analysis, the author observed that out of 25 patients with PF-induced hepatotoxicity, 22 patients discontinued the drug after detecting liver damage and received timely treatment, resulting in a favorable prognosis. However, 3 patients suffered liver toxicity that progressed to multiple organ failure and death [17].

The author conducted an comprehensive investigation into the early stages of PF-induced hepatotoxicity. Long-term toxicity experiments was arranged by a uniform design method to explore the effect of different extracts of PF on liver toxicity in rats and mice, the research results confirmed that PF has hepatotoxicity, which is related to ethanol extraction technology; alcohol extraction is more toxic than water extraction, and 70% ethanol extraction is the most toxic. Besides, there are species differences, with a more significant hepatotoxicity in mice than that in rats [18]. In the mechanism study of PF-induced hepatotoxicity, our previous research on the hepatotoxic mechanism of PF ethanol extract demonstrated that PF induces lipid droplet accumulation in mouse liver cells, thereby inhibiting the β-oxidative degradation of fatty acids in mitochondria and disrupting lipid metabolism in mice [19]. Fatty acids are essential components of lipids, and disorders in the metabolism of arachidonic acid and linoleic acid within the liver are closely associated with liver injury. Arachidonic acid is involved in glycerophospholipid metabolism and plays a significant role in pro-inflammatory signaling. The activation of arachidonic acid metabolism results in an increased production of metabolites, which can lead to metabolic disturbances in inflammatory mediators, causing the infiltration and activation of inflammatory cells in liver tissue. Furthermore, the overproduction of these metabolites may contribute to heightened oxidative stress, potentially triggering hepatocellular damage and lipid peroxidation, lead to an imbalance between lipid synthesis and oxidative decomposition, thereby exacerbating liver injury [20]. The author’s prior in vitro studies of PF-induced hepatotoxicity revealed significant apoptosis, increased intracellular lipid accumulation, elevated reactive oxygen species (ROS) levels, and reduced mitochondrial membrane potential (MMP), psoralidin demonstrating potent toxicity noteworthy [21]. In addition, in our preliminary studies, we investigated the hepatotoxic components of PF. Our findings indicate that the 70% ethanol extraction process enriches these hepatotoxic components, particularly psoralidin, bakuchiol, bavachin, isobavachin, and neobavaisoflavone. Utilizing high-content screening technology, our in vitro analysis of these enriched components demonstrated significant hepatotoxicity against L02 and HepG2 cells, with psoralidin and bakuchiol exhibiting the most pronounced toxicity. Therefore, we hypothesize that psoralidin may be one of the primary toxic constituents responsible for the hepatotoxicity associated with PF [21]. In addition, we have also noted other study indicating that psoralidin have potential hepatotoxicity [22]. However, the potential hepatotoxicity of psoralidin in animal models and the specific connection between psoralidin-induced hepatotoxicity and lipid metabolism disorders remains unclear. Clarifying this scientific issue will provide useful reference for the clinical safety of PF and the evaluation of drugs containing PF.

Based on previous findings, this study aims to investigate whether psoralidin, the primary toxic component of PF, induces hepatotoxicity in mice and to elucidate the underlying mechanisms of psoralidin-induced hepatotoxicity, with a particular focus on the balance of lipid synthesis and catabolism.

## Materials and methods

### Drug and dose design

Psoralidin, with a purity exceeding 98%, was procured from Chengdu Must Biotechnology Co., Ltd.

Regarding the dosage design, previous studies indicate that the psoralidin content in PF ranges from 0.3 to 2% [23–25]. Based on our preliminary research findings regarding the hepatotoxicity of psoralidin and considering its average content of 1.5% in PF, this study calculated the administration dosages through conversion. In accordance with the clinical dosage range for PF, three clinical doses of the herb (15, 30, and 60 g) were selected. Using the body surface area conversion method between humans and mice, the corresponding psoralidin administration doses were calculated as 38.6 mg/kg, 77.2 mg/kg, and 154.4 mg/kg, respectively.

In this study, dosages were calculated based on a psoralidin content of 1.5%, with adjustments made for the body surface area differences between humans and mice. The dosages were established as follows: 38.6 mg/kg for the low-dose group, 77.2 mg/kg for the medium-dose group, and 154.4 mg/kg for the high-dose group.

### Animals

**Fig. 1:**
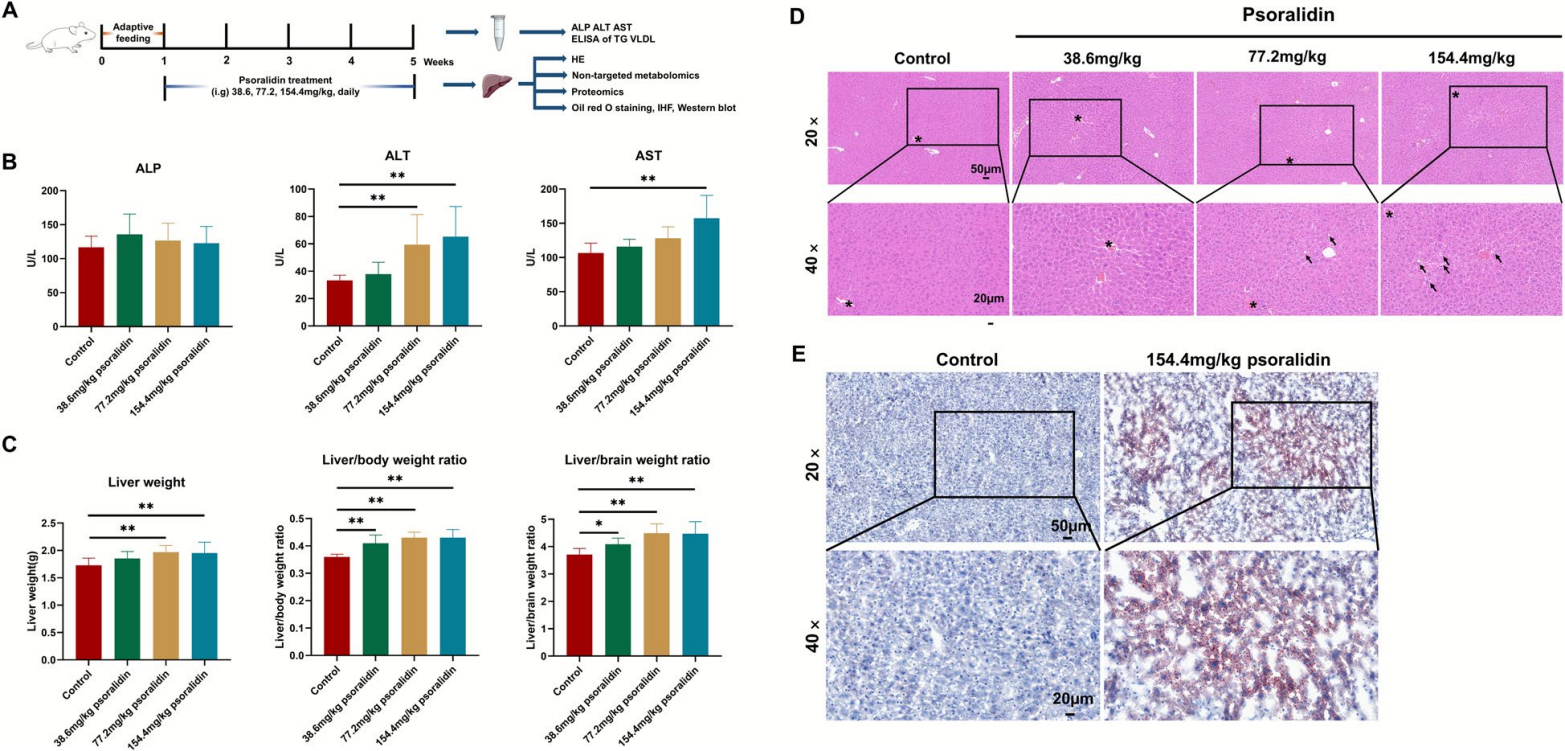
Psoralidin induced liver injury. **A** Animal experimental flowchart. **B** ALT, AST and ALP in all groups **C** Liver weight, Liver/body weight ratio, and Liver/brain weight ratio in all groups. **D** Representative histopathological microphotographs of mice liver stained with H&E at magnifications of × 20 (top line) and × 40 (bottom line). **E** Representative histopathological microphotographs of mice liver stained with oil red O staining at magnifications of × 20 (top line) and × 40 (bottom line). The hepatic lobular vein is indicated with an asterisk. Hepatocyte vacuolation is denoted with an arrow. The data are expressed as mean ± SD, *n* = 10. ^*^P < 0.05 and ^**^P < 0.01 *versus* the control group

Forty male KM mice, each weighing 30 ± 2g, were obtained from Beijing Vital River Laboratory Animal Technology Co., Ltd. (License No. SCXK (Beijing) 2021-0011). Following a 5-day acclimatization period, the mice were randomly assigned into four groups: control, psoralidin low-dose (38.6 mg/kg), medium-dose (77.2 mg/kg), and high-dose (154.4 mg/kg), with 10 animals per group. The test compounds were dissolved in a solution of 1% carboxymethylcellulose and 0.1% Tween-20 to the required concentrations for each group. Daily administration of the substances occurred at consistent intervals, while the control group received a solution of 1% carboxymethylcellulose and 0.1% Tween-20 (Fig. 1A). General physical indicators, including mobility, respiration, fur condition, and fecal characteristics, were monitored daily, with body weight recorded weekly at a consistent time. The study protocol was reviewed and approved by the Experimental Animal Ethics Subcommittee of the Academic Committee of Beijing University of Chinese Medicine under reference number BUCM-2022092205-3178.

### Biochemical assays

Blood and liver tissues were collected 12 h following the final administration. Serum was separated from the centrifuged blood to assess liver function indicators, including alanine aminotransferase (ALT), alkaline phosphatase (ALP) and aspartate aminotransferase (AST), The livers from each group were promptly excised, with the left lateral lobe divided into three sections for hematoxylin and eosin (H&E) staining, oil red O staining, and transmission electron microscopy. Western blotting assays were performed on the left medial lobe of the liver. The hepatic lobules were frozen and stored at − 80 °C for non-targeted metabolomics and proteomic analysis.

### Liver coefficient determination and histopathological examination

Following euthanizing the mice, the intact liver and brain were extracted, weighed, and organ coefficients were calculated. The liver-to-body ratio was determined by dividing the liver's wet weight by the body weight (10 g), while the liver-to-brain ratio was calculated by dividing the liver's wet weight by the brain's wet weight. For histopathological analysis, the livers from each group were subjected to fixation with 10% formaldehyde, followed by dehydration, paraffin embedding, standard sectioning, and HE staining. Pathological alterations in the liver were observed under a microscope, with scoring based on the criteria established in previous research [18].

### Oil red O staining

Liver samples from both the control group and the psoralidin high-dose group were used for further analysis. The left large lobe of the liver was rapidly frozen, followed by rewarming and drying of the frozen sections. These sections were then fixed and stained with oil red under light-proof conditions using liquid dip dye. Subsequent slight differentiation was performed with 75% alcohol, followed by hematoxylin staining and sequential sealing. Microscopic examination revealed orange-red to bright red lipid droplets and blue cell nuclei. Images were captured and analyzed for further investigation.

### Preparation of serum metabolism samples

Liver samples from the psoralidin high-dose group were thawed on ice, chopped, and thoroughly homogenized. A 20 mg portion of the sample was homogenized using a ball mill at 30 HZ, followed by centrifugation at 3000 r/min at 4 °C for 1 min. Next, 400 μL of a 70% methanol aqueous internal standard extraction solution was added, and the mixture was shaken and left to stand on ice for 15 min. After a second centrifugation at 12,000 r/min for 10 min at 4 °C, the supernatant was collected and left to stand at − 20 °C for 30 min. A final centrifugation at 12,000 r/min for 3 min at 4 °C was performed, and 200 μL of the supernatant was used for subsequent analysis.

### Non-targeted metabolomics analysis

Non-targeted metabolomics analysis was conducted using UHPLC-Q-Exactive MS, with the Waters ACQUITY UPLC HSS T3 C18 (1.8 μm, 2.1 mm × 100 mm) chromatographic column. The mobile phase consisted of (A) 0.1% formic acid solution and (B) acetonitrile. The flow rate was set to 0.4 mL/min, with a column temperature of 40 °C and an injection volume of 3 μL. The chromatographic gradient was optimized with the following parameters: 0–11 min, 95–10% A; 11.1–12 min, 10% A; 12–12.1 min, 10–95% A; 12.1–14 min, 90% A. Mass spectrometry (MS) evaluations were carried out using the Triple TOF 6600 (AB Sciex, USA). The MS conditions were as follows: for the positive ion source (ESI+), the ionization voltage was 5500 V, spray gas pressure was 50 psi, declustering voltage was 60 V, curtain gas pressure was 35 psi, and the temperature was 550 °C. For the negative ion source (ESI-), the ionization voltage was − 4500 V, spray gas pressure was 50 psi, declustering voltage was − 60 V, curtain gas pressure was 35 psi, and the temperature was 550 °C.

The raw mass spectrometry data were converted to mzXML format using ProteoWizard, followed by peak extraction, alignment, and retention time correction using the XCMS program. The 'SVR' method was employed for peak area correction, and peaks with a missing rate > 50% in each sample group were filtered out. Metabolite identification was achieved by searching the laboratory's self-built database, integrating public libraries, AI prediction libraries, and metDNA methods.

### Metabolite analysis methods

Unsupervised principal component analysis (PCA) was performed using the prcomp function in R (www.r-project.org) after data normalization through unit variance scaling. hierarchical cluster analysis (HCA) results for both samples and metabolites were visualized as heatmaps with dendrograms, while pearson correlation coefficients (PCC) between samples were calculated using the cor function in R and displayed as heatmaps. HCA and PCC analyses were carried out using the R package complex heatmap. differential metabolites between two groups were identified based on variable importance in projection (VIP) values (VIP > 1.5), *P*-values (*P* < 0.05, Student’s t-test), and fold change (FC) (FC > 1.2 or FC < 0.83). VIP values were derived from the orthogonal partial least squares discriminant analysis (OPLS-DA) results, including score plots and permutation plots, which were generated using the R package MetaboAnalystR. Prior to OPLS-DA, the data were log-transformed (log2) and mean-centered. To avoid overfitting, a permutation test with 200 permutations was conducted. The identified metabolites were annotated using the kyoto encyclopedia of genes and genomes (KEGG) Compound database (http://www.kegg.jp/kegg/compound/), and these annotated metabolites were subsequently mapped to the KEGG Pathway database (http://www.kegg.jp/kegg/pathway.html). Pathway enrichment was determined by calculating the hypergeometric test's *P*-value for the given list of metabolites.

### Preparation of liver proteomic samples

Liver samples from both the control group and the psoralidin high-dose group were quantified for protein content, followed by trypsin digestion using the filter-aided sample preparation (FASP) method. The resulting peptides were desalted using a C18 cartridge, freeze-dried, and reconstituted in a 0.1% formic acid solution for peptide quantification. Subsequently, 100 μg of peptide was subjected to tandem mass tag (TMT) labeling. Equal amounts of labeled peptides from each group were combined and fractionated using the high pH reversed-phase peptide fractionation kit. This process included column equilibration with acetonitrile and 0.1% trifluoroacetic acid, loading of the mixed labeled peptide sample, desalting with pure water through low-speed centrifugation, and binding to the column with high-pH acetonitrile solutions of increasing concentrations. The eluted peptides underwent gradient elution, followed by vacuum drying and reconstitution in 12 μL of 0.1% formic acid for peptide concentration determination.

### LC–MS/MS and proteomic analysis

The samples were separated using the easy nLC system, an HPLC system designed for nanoliter flow rates. Buffer A was a 0.1% formic acid aqueous solution, while Buffer B consisted of a 0.1% formic acid acetonitrile aqueous solution, with acetonitrile at 80%. The chromatographic column was equilibrated with a 95% A solution. Samples were injected from the autosampler into the loading column (Thermo Scientific Acclaim PepMap100, 100 μm × 2 cm, nanoViper C18) and then passed through the analytical column (Thermo Scientific EASY column, 10 cm, ID75μm, 3 μm, C18-A2) for separation at a flow rate of 300 nL/min. For DDA mode analysis, each scan cycle is consisted of one full-scan mass spectrum (R = 60 K, AGC = 300%, max IT = 20 ms, scan range = 350–1500 m/z) followed by 20 MS/MS events (R = 15 K, AGC = 100%, max IT = auto, cycle time = 2 s, TurboTMT enabled). HCD collision energy was set to 35. Isolation window for precusor selection was set to 1.2 Da. Former target ion exclusion was set for 35 s.

### Database search and bioinformatics analysis

Proteome Discoverer v1.4 software, with embedded Mascot v2.2, was utilized for database searching. The search was performed against the Swissprot_mouse_17144_20230103.fasta database, containing 17,144 sequences, along with a reverse library (decoys, Reverse database) to control the false positive rate (FDR) from random matches. TMT was employed as the quantitative method. Fixed modifications included carbamidomethyl (C), TMT6/10/16 plex (N-term), and TMT6/10/16 plex (K), while variable modifications included Oxidation (M) and TMT6/10/16 plex (Y). The peptide mass tolerance was set to 20 ppm, and the fragment mass tolerance was set to 0.1 Da. Proteins were filtered based on a peptide-level FDR of 1% to ensure result reliability. Differential protein enrichment was assessed using a two-tailed Fisher's exact test, with significance determined by a corrected *P*-value < 0.05. Protein annotation was carried out using the KAAS online tool from the KEGG database, followed by pathway mapping using the KEGG Mapper.

### Molecular docking study for targets prediction

The chemical structure of psoralidin was obtained from the PubChem website (https://pubchem.ncbi.nlm.nih.gov/). The predicted structures of potential target proteins were generated by AlphaFold Protein Structure Database(https://alphafold.ebi.ac.uk/). The protonation state of all the compounds was set at pH = 7.4, and the compounds were expanded to 3D structures using Open Babel [1]. AutoDock Tools (ADT3) were applied to prepare and parametrize the receptor protein and ligands. The docking grid documents were generated by AutoGrid of sitemap, and AutoDock Vina (1.2.0) was used for docking simulation. [2, 3] The optimal pose was selected to analysis interaction. The protein–ligand interaction figure was generated by PyMOL after docking completion.

### Transmission electron microscope observation

Liver tissues from both the control and psoralidin high-dose groups were fixed in 4% glutaraldehyde at 4 °C for 24 h, followed by post-fixation in 1% osmium tetroxide for 2 h. The tissues were then rinsed twice with PBS and subjected to a dehydration series using acetone gradients: 50% for 15 min, 70% for 15 min, 90% for 15 min, and 100% for 10 min. After dehydration, the tissues were embedded in acetone, dried, and sectioned into 70 nm slices using an ultrathin sectioning machine. These sections were mounted on copper grids, stained with uranyl acetate and lead citrate for 30 min, and examined under a transmission electron microscope.

### Immunofluorescence analysis

The level of ROS in liver was measured using immunofluorescence analysis. The paraffin sections were subjected to permeabilisation, sealing, primary antibody incubation, fluorescent secondary antibody incubation, re-staining of nuclei and sealing operations, and then the expression of ROS was observed by fluorescence microscopy.

### Enzyme-linked immunosorbent assay (ELISA)

Serum triglycerides (TGs) and very low-density lipoproteins (VLDL) were quantified using an ELISA kit according to the manufacturer’s instructions.

### Western blotting

For liver tissue analysis, proteins were extracted and quantified using a BCA assay. The extracted proteins were separated by 12% SDS-PAGE and transferred to PVDF membranes. The membranes were blocked with 5% skim milk in TBST for 60 min at room temperature. Dilute each primary antibody using a universal antibody diluent at the specified ratios: anti-Acot4 (1:1000), anti-Pla2g6 (1:1000), anti-Pla2g12b (1:1000), anti-Plin5 (1:2000), anti-Cyp4a10 (1:1000), anti-Cyp2u1 (1:1000) and anti-Cytochrome C (1:1000). After the blocking step, add 5 mL of the appropriately diluted primary antibody to each protein sample and incubate at 4 °C for 12 h. After washing with TBST, secondary antibodies were applied for 1 h at room temperature. The membranes were subsequently washed and analyzed using the Amersham Imager 680 UV system for exposure and grayscale value analysis.

### Statistical analysis

Experimental data were expressed as Mean ± SD. Statistical analyses were performed using GraphPad Prism 8.0 software, employing the One-way ANOVA method. The LSD method was applied for equal variances, while Dunnett's T3 method was used for unequal variances. Statistical significance was defined as *P* < 0.05, with a highly significant difference indicated by *P* < 0.01.

## Results

### Psoralidin-induced hepatotoxicity in mice

No significant administration-related changes were observed in the control group. However, in the medium-dose psoralidin group, a significant increase in ALT levels was detected (*P* < 0.01), while the high-dose group exhibited significant elevations in both ALT and AST levels (*P* < 0.05, *P* < 0.01) (Fig. 1B). ALT and AST are enzymes typically localized within cells and are released into the extracellular space upon cell damage. These findings confirmed that psoralidin at medium and high doses caused hepatocellular damage in mice.

The low-dose psoralidin group demonstrated a notable increase in both liver-to-body and liver-to-brain ratios in mice (*P* < 0.05, *P* < 0.01). Furthermore, the medium-dose and high-dose groups showed significant increases in liver weight, liver-to-body ratio, and liver-to-brain ratio (*P* < 0.01). Notably, the high-dose group exhibited pronounced hepatic injury, with a 22.2% increase in liver-to-body ratio and a 20.5% increase in liver-to-brain ratio (Fig. 1C).

To further elucidate the potential hepatotoxic effects of psoralidin, histopathology analyses were conducted. As shown in Fig. 1D, psoralidin at a medium dose induced hepatocyte hypertrophy and the degeneration of fat vesicles and microvesicles in 40% of the mice. In the high-dose group, approximately 60% of the mice exhibited similar hepatocyte hypertrophy and fat vesicle degeneration. These results underscore a dose-dependent relationship between psoralidin administration and the incidence and severity of hepatotoxicity, consistent with previous research on the effects of PF ethanol extract on liver tissue (Fig. 1D). This study confirmed that psoralidin is a significant toxic component of PF. And the oil red O staining results confirmed the presence of significant hepatic steatosis in the PF group of mice. As shown in Fig. 1E, no fat droplet aggregation was observed in the liver tissue of the control group, whereas the high-dose psoralidin group exhibited significant red fat droplet accumulation. This is consistent with the HE results, which suggested that psoralidin-induced hepatotoxicity linked to liver fat accumulation.

### Untargeted metabolomics analysis indicated that psoralidin inhibited fatty acid metabolism and induced hepatic lipotoxicity in mice

**Fig. 2:**
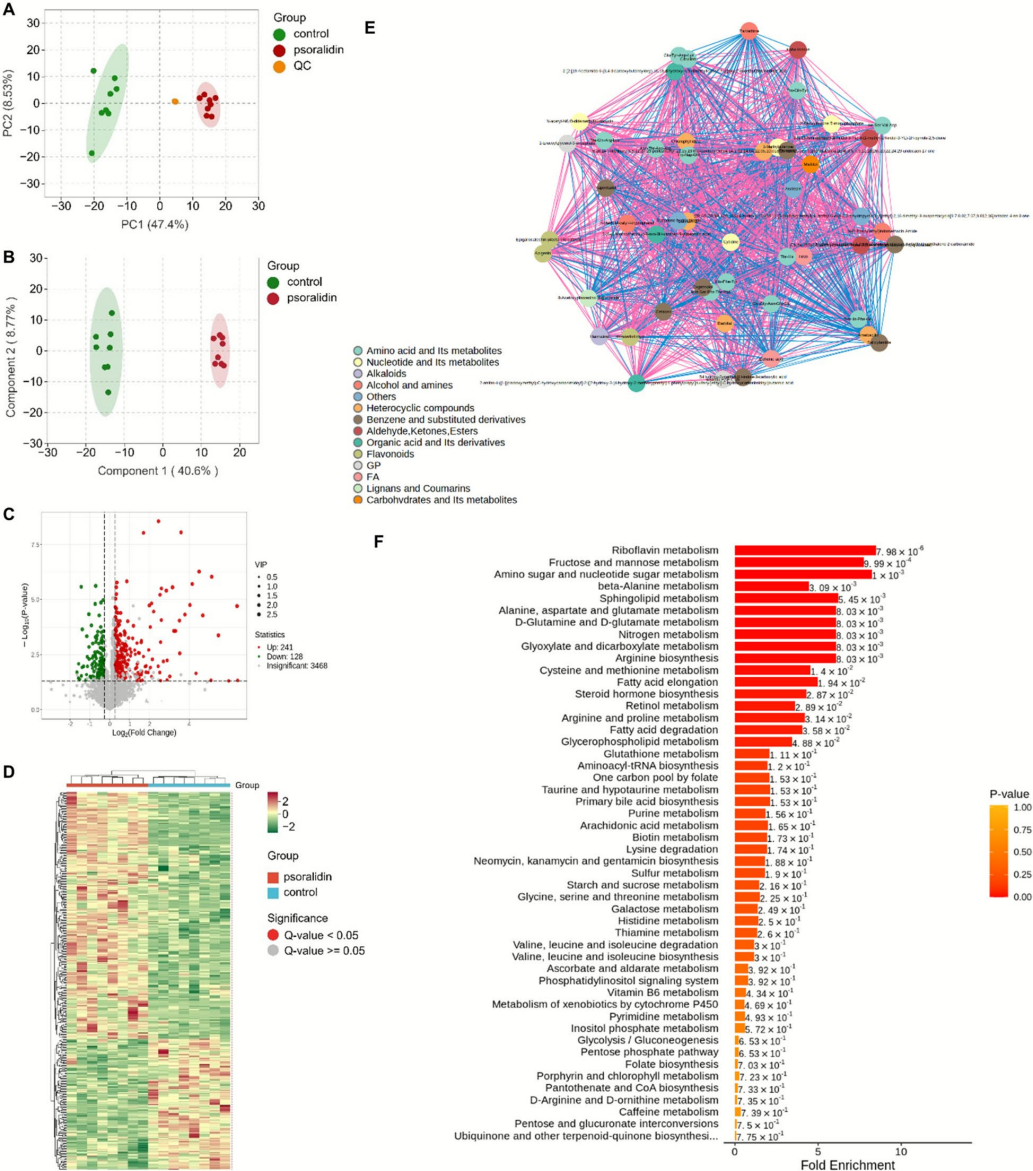
Metabolomic analysis of mice liver tissues after psoralidin administration. **A** PCA scores. **B** OPLS-DA scores. **C** Volcano plot. Red and green dots indicate significantly increased and decreased proteins, respectively. *P* < 0.05, VIP > 1.2, FC > 1.2, or < 0.5. **D** The heat map shows 369 metabolites that were substantially altered in the liver were clustered in the control and psoralidin group. The red and green represent increased and decreased metabolite content, respectively. **E** Correlation between the top 50 VIP differential metabolites. **F** The KEGG pathway classification of differential proteins in the PFE group

To elucidate the mechanism of psoralidin-induced hepatotoxicity in mice, a untargeted metabolomics analysis was conducted. A total of 5,496 metabolites were detected, with 3,387 identified at the MS 2 level, which were the focus of subsequent analyses. Figure 2A shows a clear separation of samples between the control group and the psoralidin-treated group, indicating significant metabolic alterations post-psoralidin intervention. The metabolomic data were further analyzed using the OPLS-DA model, where subgroup scores highlighted the notable differences between the psoralidin and control groups (Fig. 2B).

Psoralidin administration led to significant changes in 369 differential metabolites at the MS 2 level, characterized by a VIP > 1, *P*-value < 0.05, and FC > 1.2 or < 0.5. Among these, 241 metabolites were upregulated, including phosphocholine, LPC(18:0/0:0), and hexanoyl-coenzyme A, while 128 metabolites were significantly downregulated, such as diethanolamine (Fig. 2C). Detailed information on the retention time, metabolite formulas, *P*-values, and fold changes for these differential metabolites is provided in Supplementary Table S1. To visualize changes in relative metabolite content, a heatmap was generated using the R software ComplexHeatmap package (Fig. 2D). The heatmap demonstrates that samples within the same group clustered effectively, with distinct differences in metabolite levels between the control and psoralidin groups, indicating significant alterations in the mice's metabolism following psoralidin intervention. Moreover, correlations among the top 50 VIP differential metabolites revealed associations between amino acids, fatty acids, carbohydrates, and their related metabolites (Fig. 2E).

Using information from the KEGG database, topology and pathway enrichment analyses were conducted to identify the metabolomic pathways involved. The identified biomarkers and related pathways are presented in Fig. 2F. The findings suggest that psoralidin-induced hepatotoxicity is significantly associated with alterations in pathways including unsaturated fatty acid biosynthesis, glycerophospholipid metabolism, fat digestion and absorption, fatty acid degradation, fatty acid metabolism, alpha-linolenic acid metabolism, arachidonic acid metabolism, the tricarboxylic acid cycle, oxidative phosphorylation, cholesterol metabolism, 2-oxocarboxylic acid metabolism, glutathione metabolism, and sphingomyelin metabolism.

### Proteomic analysis revealed that abnormalities in lipid synthesis and metabolism are associated with psoralidin-related hepatotoxicity

**Fig. 3:**
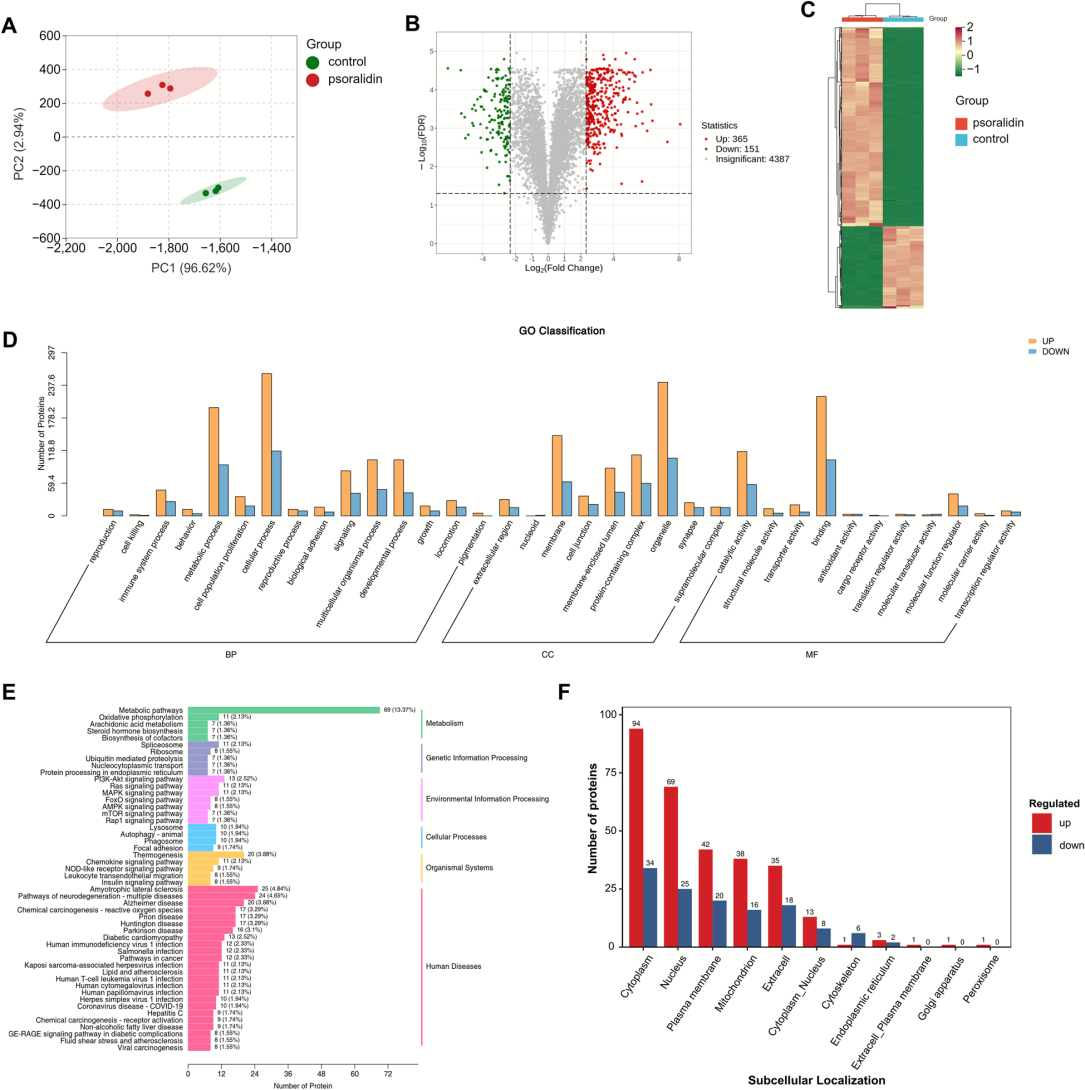
Proteomic analysis of mice liver tissues after psoralidin administration. **A** PCA scores. **B** Volcano plot. Green and red dots represent significantly decreased and increased proteins, respectively. *P* < 0.05, FC > 5 or < 2. **C** The heat map shows that 516 proteins that were substantially altered in the liver were clustered in the control and psoralidin groups. The red and green represent increased and decreased metabolite content, respectively. **D** GO analysis of differential proteins in the psoralidin group. **E** KEGG pathway analysis of differential proteins changed in the psoralidin group. **F** Subcellular localization analysis of differential proteins

Proteomics analysis was conducted to explore potential targets and pathways associated with psoralidin-induced hepatotoxicity, leading to the identification of 4,903 proteins. Principal component analysis revealed a clear separation between the control group and the psoralidin-treated group, indicating significant differences in protein expression profiles between these groups (Fig. 3A). A total of 516 differential proteins were identified based on the screening criteria of FC > 5 or FC < 0.2 with a *P*-value < 0.05. The extent of differences for each differential protein is visualized in Fig. 3B. Among these, 365 proteins were upregulated, and 151 proteins were downregulated in the psoralidin-treated group compared to the control group. Detailed information on these differential proteins is provided in Table S2 of the Supplementary Material.

Z-score normalization of the differential proteins followed by clustering heatmap generation revealed significant variations in protein levels between the psoralidin and control groups, indicating substantial changes in protein expression following psoralidin administration (Fig. 3C). GO annotation classification analysis highlighted the involvement of these differential proteins in various biological processes, cellular components, and molecular functions (Fig. 3D).

KEGG pathway analysis identified several biological pathways linked to complement-induced liver injury in mice. Differential proteins associated with psoralidin-induced hepatotoxicity were found to be involved in pathways such as oxidative phosphorylation, arachidonic acid metabolism, autophagy, and the biosynthesis of unsaturated fatty acids, as shown in Fig. 3E. Furthermore, subcellular localization analysis indicated that these differential proteins were predominantly localized in the cytoplasm, nucleus, plasma membrane, and mitochondria (Fig. 3F).

### Psoralidin induced abnormalities in biosynthesis of unsaturated fatty acid, fatty acid metabolism, arachidonic acid metabolism and phospholipid metabolism

**Fig. 4:**
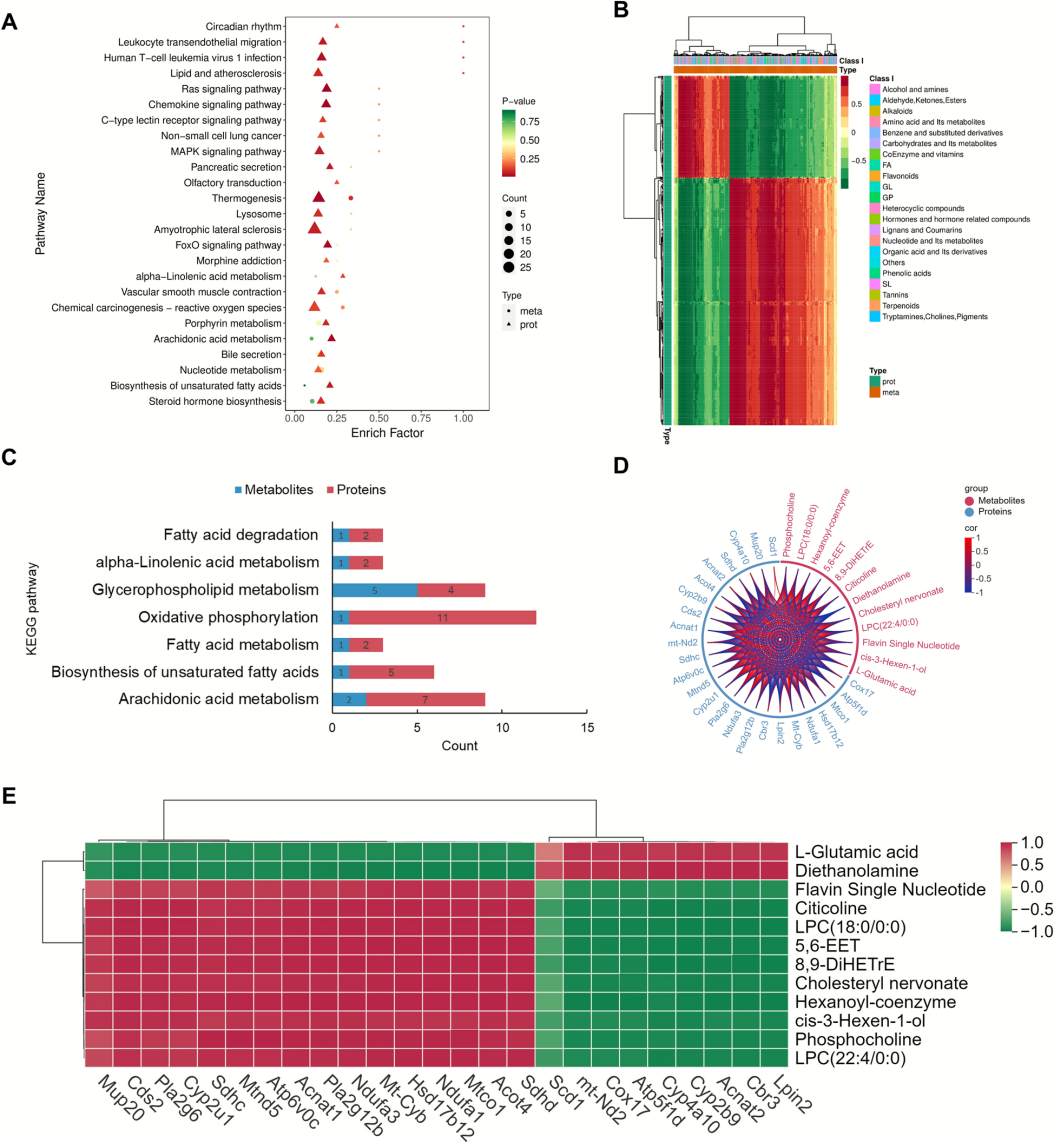
Association analysis of metabolomics and proteomics. **A** The top 25 KEGG pathways in both metabolomics and proteomics based on *P*-value. **B** A cluster analysis on the differential proteins and metabolites within the metabolomics and proteomics shared pathways. **C** Pathway screening for potential hepatotoxicity mechanisms. **D** Chord diagram for metabolites and proteins association analysis. **E** Correlation analysis of key metabolites and proteins

Following the KEGG enrichment analysis of metabolites and proteins, 138 pathways were identified as commonly enriched by both histologies. Among these, the top 25 pathways, ranked by *P*-value and shown in Fig. 4A, included thermogenesis, arachidonic acid metabolism, unsaturated fatty acid biosynthesis, and α-linolenic acid metabolism. Differential proteins and metabolites within these shared metabolomics and proteomics pathways underwent cluster analysis. As depicted in Fig. 4B, the classification of these differential proteins and metabolites covered a range of categories, including fatty acids, ketones, esters, amino acids and their metabolites, carbohydrates and their metabolites, tryptophan, and choline.

Previous studies on the hepatic toxicity mechanism of PF highlighted a strong association with pathways such as oxidative phosphorylation, unsaturated fatty acid biosynthesis, fatty acid metabolism, cholesterol metabolism, and arachidonic acid metabolism. In the current investigation into psoralidin-induced hepatotoxicity, significant alterations were observed in the pathways of biosynthesis of unsaturated fatty acid, fatty acid metabolism, arachidonic acid metabolism, phospholipid metabolism, and oxidative phosphorylation (Fig. 4C). These findings align with previous research, implying that these pathways may be pivotal in psoralidin-induced hepatotoxicity. Subsequent correlation analysis of the differential metabolites and proteins enriched in these pathways, as shown in Fig. 4D, revealed 12 differential metabolites, such as flavin mononucleotide and L-glutamic acid, which were directly correlated with 25 differential proteins including phospholipase A2, group VI (Pla2g6), phospholipase A2, group XIIB (Pla2g12b), cytochrome P450, family 4, subfamily a, polypeptide 10 (Cyp4a10), and cytochrome P450 2U1 (Cyp2u1). Figure 4E demonstrates the correlation between these differential proteins and metabolites, highlighting a positive correlation between Cyp2u1 and flavin mononucleotide and a negative correlation between Pla2g12b and L-glutamic acid.

### Screening for potential targets of psoralidin-induced hepatotoxicity

**Fig. 5:**
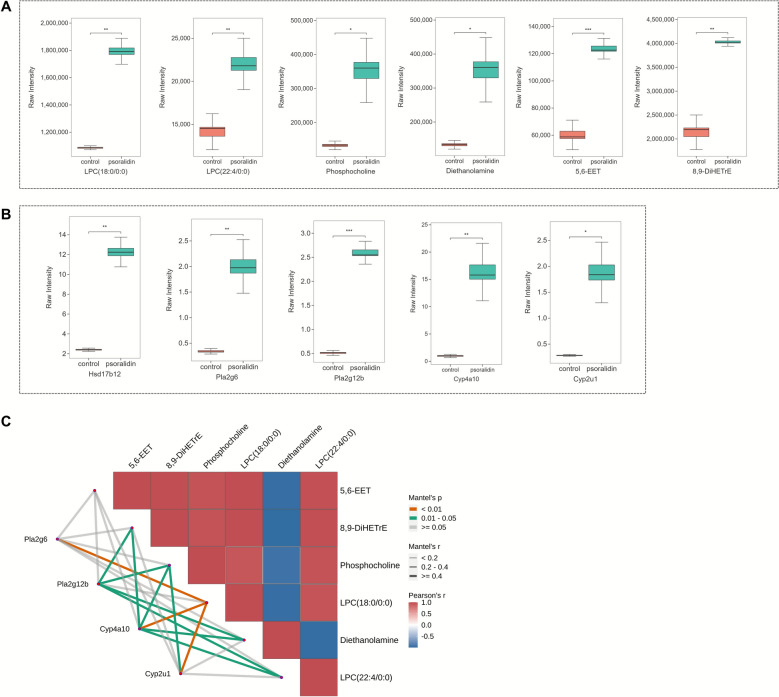
Analysis of metabolites and proteins related to the mechanism of psoralidin hepatotoxicity. **A** Box plot of key metabolites. **B** Box plot of key proteins. **C** Pearson analysis of key metabolites and proteins

Further analysis determined that psoralidin intervention led to significant changes in metabolites within the potential pathways identified earlier, including lysophosphatidylcholine (LPC)(18:0/0:0), LPC(22:4/0:0), phosphocholine, diethanolamine, 5,6-EET, and 8,9-DiHETrE (Fig. 5A). Notably, proteins such as 7β-Hydroxysteroid dehydrogenases type 12 (HSD17B12), Pla2g12b, Cyp2u1, and Cyp4a10 were involved in multiple lipid metabolism-related pathways, playing roles in various lipid metabolism processes with significantly different raw intensities (Fig. 5B). These observations suggest that these proteins could serve as potential targets of psoralidin-induced liver injury. Pearson correlation analysis of these significant metabolites and proteins further indicated a strong regulatory relationship between them (Fig. 5C), suggesting that these proteins may regulate the expression of these metabolites and could be potential targets for psoralidin-induced hepatotoxicity.

### Molecular docking

**Fig. 6:**
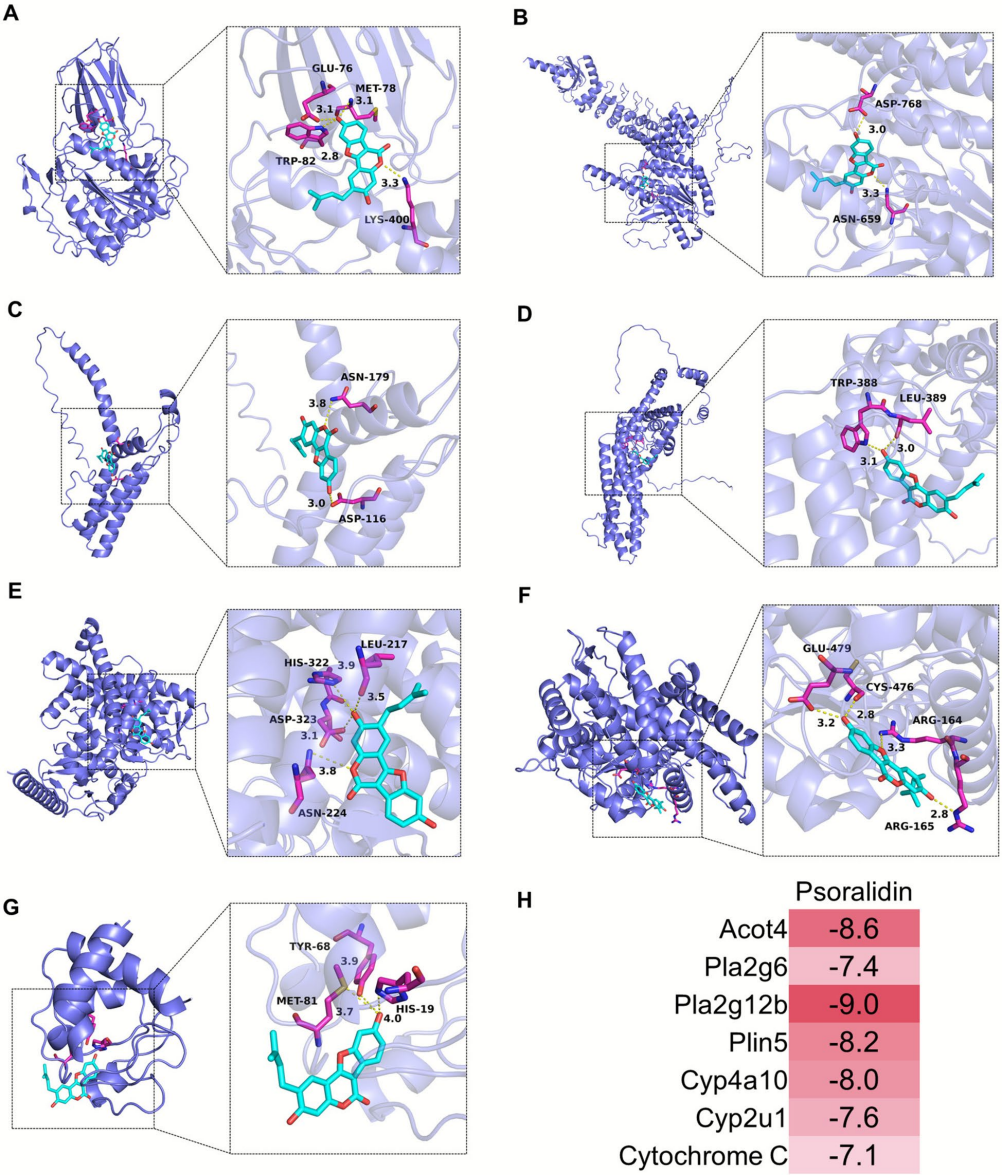
The molecular docking models of targets and components. **A** Acot4; **B** Pla2g6; **C** Pla2g12b; **D** Plin5; **E** Cyp4a10; **F** Cyp2u1; **G** Cytochrome C; **H** Binding activity between the target proteins and psoralidin

Based on the results of multi-omics correlation analysis, Pla2g6, Pla2g12b, Cyp4a10, Cyp2u1 were selected for molecular docking analysis with psoralidin; in addition, Acot4 and Plin5, which is closely related to lipid synthesis, and Cytochrome C, which is related to mitochondrial damage, were selected for molecular docking simulation with psoralidin. The results of molecular docking indicated the binding activity between the receptor and ligand in terms of the magnitude of the binding energy, with smaller binding energies indicating higher affinity between the receptor and ligand, binding energies < − 5.0 kJ/mol indicating higher binding activity, and binding energies < − 7.0 kJ/mol indicating very strong binding activity[26]. We observed that the binding energies of Acot4, Pla2g6, Pla2g12b, Plin5, Cyp4a10, Cyp2u1and Cytochrome C with psoralidin were − 8.6 kcal/mol, − 7.4 kcal/mol, − 9.0 kcal/mol, − 8.2 kcal/mol, − 8.0 kcal/mol, − 7.6 kcal/mol and − 7.1 kcal/mol, respectively, suggesting that 7 potential targets have strong binding energies with psoralidin, their 3D binding structures were shown in Fig. 6. Overall, the ligands and receptors were bound mainly by hydrogen bonds.

### Psoralidin inhibited lipid metabolism and induces hepatic lipid deposition and mitochondrial dysfunction

**Fig. 7:**
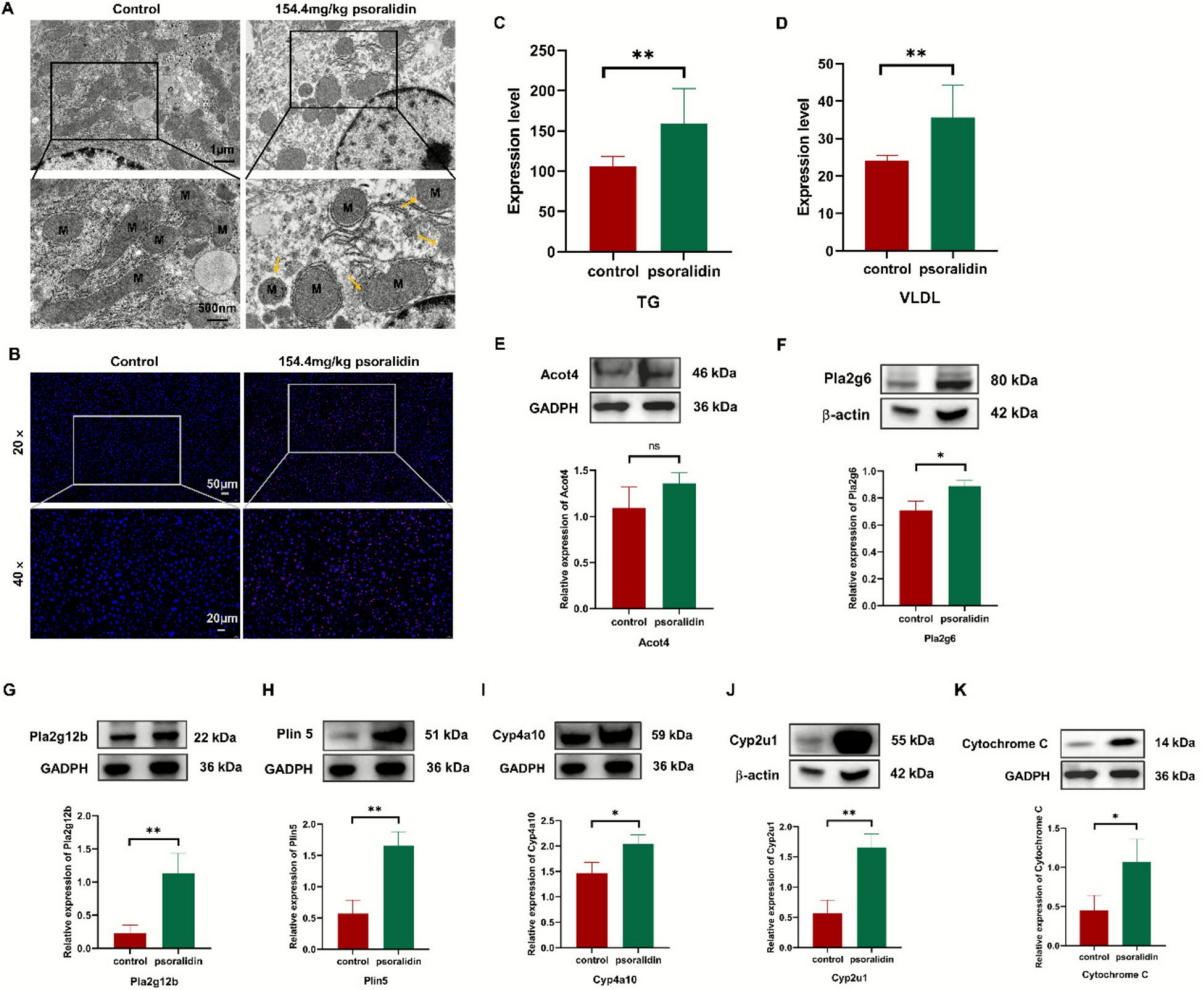
Psoralidin interferes with lipid metabolism in mice's liver. **A** The ultrastructural changes of the liver mitochondria by transmission electron microscope, Scale bars = 500 μm. M denotes mitochondria, yellow arrow indicate the sites of mitochondrial damage. **B** ROS in the liver. **C** TGs in the serum. **D** VLDL in the serum. **E–K** Western blot analysis of Acot4, Pla2g6, Pla2g12b, Plin5, Cyp2u1, Cyp4a10, and Cytochrome C expressions in the liver of mice. The values were expressed as mean ± SD, *n* = 3. ^*^P < 0.05, ^**^P < 0.01 *vs*. control group

An imbalance between fat synthesis and oxidative breakdown can lead to lipid metabolism disorders. To investigate whether psoralidin promotes lipid accumulation by enhancing lipid synthesis and inhibiting oxidative catabolism, hepatic lipid distribution and mitochondrial morphology were examined, alongside proteomics-derived indices related to lipid synthesis and oxidative catabolism. As shown in Fig. 7A, transmission electron microscopy revealed a reduction in mitochondrial number, blurred mitochondrial boundaries, and disorganized mitochondrial cristae in the liver tissue of the high-dose psoralidin group, pointing to a potential connection between psoralidin hepatotoxicity and mitochondrial dysfunction. In addition, psoralidin induced elevated ROS levels in liver, suggesting that lipid accumulation may be related to lipid peroxidation caused by elevated ROS (Fig. 7B). Serum analysis further demonstrated that psoralidin significantly increased TGs and VLDL levels in mice. These results suggest that psoralidin induced hepatic steatosis may be driven by TGs accumulation (Fig. 7C, D). Based on these data, and in conjunction with histopathological findings, we hypothesize that the hepatotoxicity induced by psoralidin is associated with lipid metabolism disorders caused by mitochondrial damage.

To further elucidate the key targets of psoralidin-induced hepatotoxicity, western blotting analysis showed that psoralidin exposure markedly upregulated the expressions of Acot4 (Fig. 7E), which in turn lead to increased levels of TGs and free fatty acids (FFAs) in mice. Additionally, psoralidin catalyzed the conversion of phosphatidylcholine into LPC by enhancing the expressions of Pla2g6 and Pla2g12b (Fig. 7F, G), which promoted the synthesis and accumulation of TGs. In addition, up-regulation of Plin5, Cyp4a10, Cyp2u1 results in excess ROS being released (Fig. 7H–J), and elevated Cytochrome C level indicated mitochondrial damage in mouse livers (Fig. 7K). All these results confirm that psoralidin could both induce lipid deposition and inhibit lipid oxidative decomposition, thereby inducing hepatic lipotoxicity.

## Discussion

**Fig. 8:**
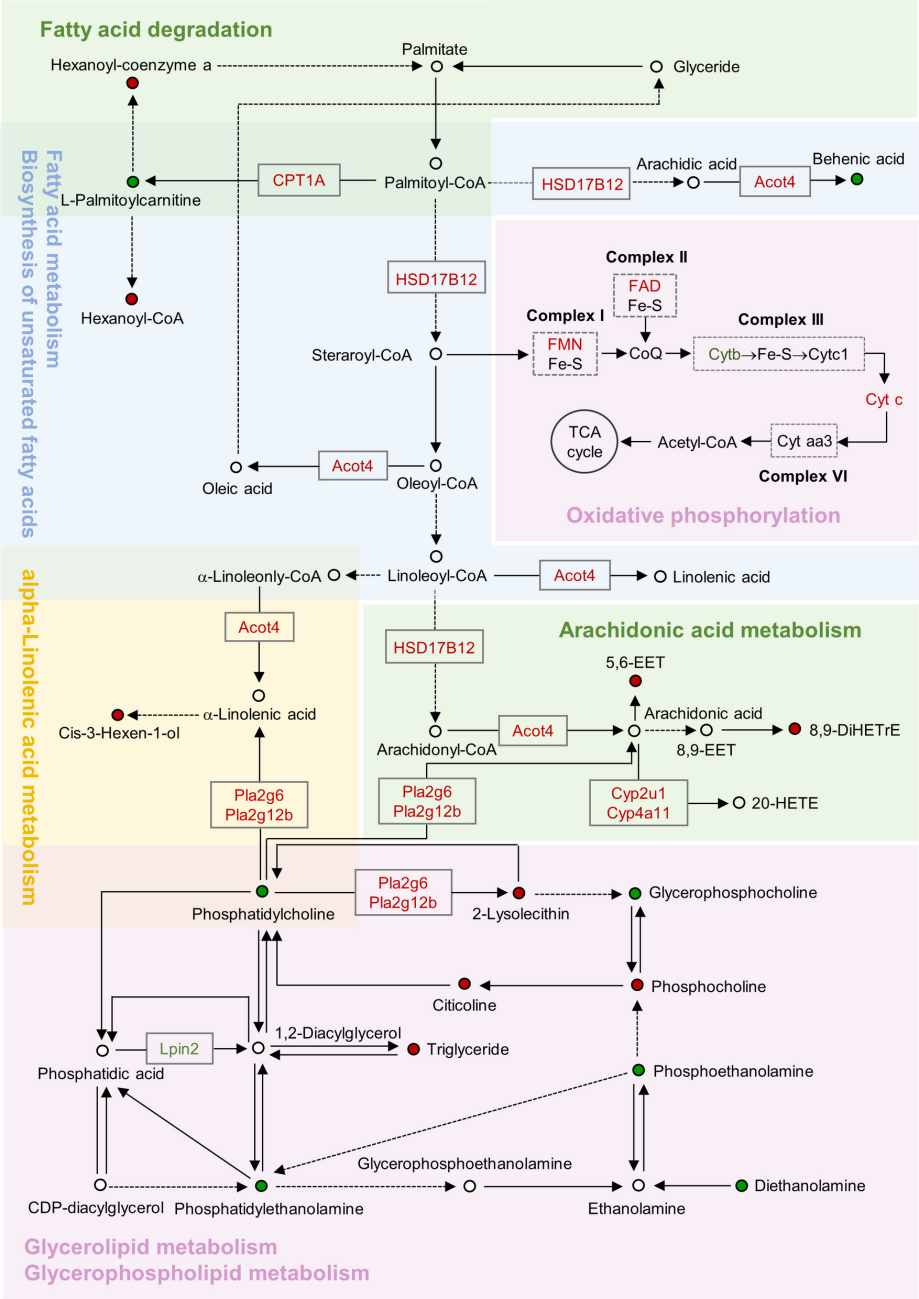
Psoralidin caused hepatotoxicity by inducing lipid biosynthesis and impeding lipid oxidative catabolism to caused disruption of lipid synthesis-catabolism homeostasis

This study conducted a thorough investigation into the toxicity confirmation and mechanisms of psoralidin at the animal, organ, and molecular levels. The long-term toxicity experiment conducted on mice demonstrated that psoralidin induced both microvesicular and macrovesicular steatosis, along with lipid accumulation in the liver at the organ level. Regarding mechanism exploration, unlike previous studies, we demonstrated that psoralidin induces fat deposition via promoting lipid synthesis and inhibiting lipid oxidative decomposition. On the one hand, psoralidin induced the expression of Acot4 and Plin5, which in turn lead to up-regulations of TGs and FFA in mice, and increased the HSD17B12 level, thereby promoting the synthesis of long-chain fatty acids and facilitating lipid synthesis. Additionally, psoralidin catalyzed the conversion of phosphatidylcholine into LPC by enhancing the expressions of Pla2g6 and Pla2g12b, which promoted the synthesis and accumulation of TGs, ultimately inducing disorders in glycerophospholipid metabolism. on the other hand, psoralidin also caused mitochondrial damage, leading to a decrease in FA oxidation, resulting in the accumulation of a large number of lipid droplets in the liver. These findings indicate an imbalance between lipid synthesis and degradation, resulting in excessive lipid accumulation in the liver, which ultimately manifests as hepatotoxicity (Fig. 8).

In terms of lipid synthesis, the liver as the central hub for lipid metabolism, disruptions in hepatic lipid metabolism lead to non-physiological TGs accumulation, known as steatosis. Hepatic steatosis can increase fatty acid intake, enhance fat synthesis and TGs accumulation, reduce fatty acid oxidation and impaire secretion of TGs or VLDL [27]. The liver, as the primary site for TGs synthesis, secretes TGs mainly as VLDL into the bloodstream for utilization [28]. Under conditions of elevated extracellular fatty acid concentrations, if TGs synthesis exceeds the secretion capacity, triacylglycerol accumulates within cells, leading to tissue steatosis [29]. Acot4 is a key enzyme in fatty acid activation, unsaturated fatty acid biosynthesis, and fatty acid β-oxidation. Overexpression of Acot4 leads to elevated levels of TGs and FFA in mice, thereby promoting lipid synthesis [30]. Moreover, HSD17B12 can induce fatty acid elongation, which is critical for lipid formation[31–33]. In line with these findings, this study confirmed that psoralidin increased the TGs and VLDL levels, and up-reglulated the Acot4 and HSD17B12, which were significantly enriched in fatty acid elongation and biosynthesis of unsaturated fatty acid, suggested that the elevations of Acot4 and HSD17B12 enhance TGs synthesis in the liver, induce the synthesis of long-chain fatty acids and contribute to hepatic steatosis.

Perilipin 5 (Plin 5) is a member of the Perilipin family of lipid droplet coat proteins, primarily located on the surface of lipid droplets. As a cytoplasmic lipid droplet protein, Plin 5 coordinates the actions of lipases at the lipid droplet surface and may mediate interactions between lipid droplets and mitochondria. In cells, Plin 5 plays an essential role in maintaining intracellular lipid balance and metabolic homeostasis by promoting TGs storage and/or reducing fatty acid oxidation, thereby promoting overall lipid accumulation within the cell [34–36]. Moreover, Plin 5 serves as a crucial linker protein for anchoring lipid droplets to mitochondria, where physical connections are formed through specific protein or molecular interactions. This contact is vital for facilitating the transport of fatty acids from lipid droplets to mitochondria for oxidative energy production and signal transduction [37]. Plin 5 is present at lipid droplet-mitochondria contact sites, and its overexpression induces the recruitment of mitochondria to the periphery of lipid droplets, forming peridroplet mitochondria, while bidirectionally regulating lipid metabolism to maintain homeostasis [38, 39]. Metabolically, Plin 5 stabilizes lipid droplets by inhibiting their hydrolysis and directing fatty acids towards triglyceride synthesis and storage, while downregulating the expression of carnitine palmitoyltransferase-1 (CPT-1) and genes related to mitochondrial oxidation to restrict fatty acid oxidation in mitochondria, thereby promoting overall intracellular lipid accumulation. To further investigate the cell-autonomous function of Plin 5 in lipid metabolism, our proteomic studies revealed that Plin 5 expression was significantly upregulated in mouse liver following psoralen intervention. Subsequently, we confirmed through Western blot analysis that psoralidin induced the expression of Plin 5. Based on previous research findings, we hypothesize that Plin 5 overexpression promotes the contact between mitochondria and lipid droplets. However, psoralidin damages mitochondrial structure, leading to reduced mitochondrial oxidative function. This results in the accumulation of a large number of lipid droplets in the liver that cannot be oxidized and degraded, ultimately inducing hepatic steatosis.

Notably, our study also found that both Pla2g6 and Pla2g12b were up-regulated and involved in the regulation of α-linolenic acid metabolism, linoleic acid metabolism, glycerophospholipid metabolism and arachidonic acid metabolism. The phospholipase A2 (PLA2) hydrolyzes the ester-acyl bond at the second position of phospholipids, releasing free fatty acids and lysolecithin, predominantly arachidonic acids (AAs). On the one hand, once AAs is released from the cell membrane via PLA2, the CYP450 metabolic pathway becomes the primary route for its metabolism in the liver [40]. CYP4A enzyme is integral to the metabolism of both AAs and medium-chain fatty acids. However, CYP4A can catalyze cycle uncoupling, leading to excessive production of ROS, which may exacerbate disease progression [41]. In this study, we also confirmed that An increase in Cyp4a10 enhances the oxidation of fatty acids in microsomes, leading to an imbalance in the body's oxidation-antioxidant equilibrium. This imbalance may result in heightened oxidative stress and lipid peroxidation in the liver, generating excessive ROS and lipid peroxides. On the other hand, iPLA2β catalyzes the degradation of phosphatidylcholine (PC) to LPC, which can promote TGs accumulation [42, 43]. Pla2g12b has been identified as a novel regulator of TGs metabolism in the liver, facilitating hepatic VLDL-TGs secretion [44]. Our metabolomics analysis revealed significant increases in phosphocholine, LPC(18:0/0:0), LPC(22:4/0:0) and citicoline levels within the glycerophospholipid metabolism pathway. Dysregulated metabolism of these glycerophospholipids lead to the accumulation of potentially lipotoxic lipids, such as saturated fatty acids, LPC, ceramide, sphingolipids and diacylglycerols, which can damage lipid cells [45, 46]. Abnormal phospholipid synthesis disrupts glycerophospholipid metabolism, leading to increased accumulation in the liver and a reduced capacity to eliminate significant amounts of endogenous toxins. The findings of this research suggested that psoralidin induces hepatic lipid metabolism disorders in mice by disrupting glycerophospholipid metabolism homeostasis. This disruption is mediated through the upregulations of Pla2g6 and Pla2g12b, which promote TGs synthesis and accumulation while catalyzing phosphatidylcholine into LPC. The accumulations of phospholipid metabolites subsequently induce glycerophospholipid metabolism disorders, contributing to hepatic steatosis.

In lipid metabolism, lipid droplets (LDs) serve as crucial organelles for lipid storage within cells, whereas mitochondria function as the energy factories responsible for energy metabolism and oxidative phosphorylation. The interaction between intracellular LDs and mitochondria is crucial for regulating lipid metabolism and maintaining energy balance[37]. Plin 5 anchors mitochondria to the lipid droplet membrane via the outermost region of its carboxyl terminus, and its overexpression promotes the recruitment of mitochondria to the periphery of LDs. Metabolically, Plin5 stabilizes LDs by inhibiting their hydrolysis and directing the synthesis and storage of fatty acids (FAs) as TGs, thereby limiting the mitochondrial oxidation of FAs[38, 47, 48]. In this study, psoralidin induced the overexpression of Plin5, which facilitated the generation of LDs and enhanced the contact between LDs and mitochondria, resulting in the accumulation of a significant number of lipid droplets in the liver. Concurrently, psoralidin also caused mitochondrial damage, leading to a decrease in FAs oxidation. Collectively, these outcomes heightened hepatic lipotoxicity, ultimately resulting in hepatotoxicity.

In summary, a research framework that focuses on toxic components, target organs, toxicity pathways and toxicity targets was carried out to explore the psoralidin-induced liver injury. In the validation of psoralidin’s hepatotoxicity, long-term animal experiments and multi-omics analysis methods have confirmed that psoralidin is one of the primary hepatotoxic components of PF. This aligns with earlier pathological observations on the hepatotoxicity of PF extract, verifing psoralidin as a principal toxic component of PF. These results may inform future revisions of toxic ingredient limits in PF medicinal standards, potentially leading to the classification of psoralidin as a regulated toxic ingredient. Furthermore, we would like to emphasize that psoralidin induces hepatotoxicity through a dual intervention mechanism. On one hand, it promotes lipid biosynthesis, resulting in lipid deposition; on the other hand, it impairs mitochondrial function, thereby hindering lipid oxidation and degradation. This disruption ultimately disturbs the balance between lipid synthesis and catabolism.

This study highlights that, building on previous research, it focuses on disorders of lipid metabolism. It confirms that psoralidin promotes lipid synthesis and inhibits lipid metabolism from both the perspectives of lipid synthesis and metabolism. This disruption of the balance between lipid synthesis and metabolism leads to lipid deposition and liver toxicity. The highlights of this study are as follows: firstly, it underscores the hepatotoxicity of PF for clinical medication safety, emphasizing the necessity of regular liver function monitoring during PF consumption and cautioning against excessive intake. Secondly, the study highlights the importance of being vigilant regarding the potential toxicity of Chinese herb throughout the drug development process, thereby offering references for the development and evaluation of new drugs containing PF. Thirdly, this study confirms that psoralidin is one of the main hepatotoxic components of PF. The findings suggest that in the revision of the quality standards for PF, attention should be paid to the content of toxic components such as psoralidin, which will help promote the improvement of the standards related to PF.

This study acknowledges certain limitations. In our preliminary research on the hepatotoxicity of PF, we conducted long-term toxicity experiments using both Sprague–Dawley (SD) rats and KM mice. The results revealed species-specific differences in the hepatotoxicity of PF, with KM mice exhibiting more pronounced liver toxicity. Consequently, we selected KM mice as the primary animal model for subsequent studies. However, we did not perform comparative analyses between KM mice and other strains, such as C57BL/6 mice. Future research will focus on conducting comprehensive studies on the mouse models of hepatotoxicity associated with PF.

## Conclusions

This study has demonstrated psoralidin is one of the hepatotoxic components of PF, which induces liver injury via promoting lipid synthesis, inhibiting lipid oxidative decomposition, and regulating pathways of phospholipid metabolism.

## Supplementary Information

Additional file 1.

Additional file 2.

## Data Availability

The data supporting this study’s findings are available from the corresponding author upon reasonable request.

## Abbreviations

AA: Arachidonic acid
Acot4: Acyl CoA thioesterase 4
ALP: Alkaline phosphatase
ALT: Alanine transaminase
AST: Aspartate transaminase
CPT1A: Carnitine palmitoyl transferase 1a
Cyp2u1: Cytochrome P450 2U1
Cyp4a10: Cytochrome P450 A10
ELISA: Enzyme-linked immunosorbent assay
FC: Fold change
FFA: Free fatty acids
H&E: Hematoxylin and eosin
HSD17B12: 7β-Hydroxysteroid dehydrogenases type 12
KEGG: Kyoto Encyclopedia of Genes and Genomes
LPC: Lysophosphatidylcholine
MMP: Mitochondrial membrane potential
OPLS-DA: Orthogonal partial least squares discriminant analysis
PC: Phosphatidylcholine
PCA: Principal component analysis
PCC: Pearson correlation coefficients
PF: Psoraleae Fructus
PLA2: Phospholipase A2
Pla2g12b: Phospholipase A2 group XIIB
Pla2g6: Phospholipase A2 group VI
Plin5: Perilipin 5
ROS: Reactive oxygen species
TCM: Traditional Chinese medicine
TGs: Triglyceride
TMT: Tandem Mass Tag
VIP: Variable importance in projection
VLDL: Very low-density lipoproteins
