# Supplementary material for: Psoralidin, a main compound in Psoraleae Fructus, induces hepatotoxicity by impeding lipid oxidative catabolism and aggravating lipid accumulation in mice
Source: Chin Med. 2026 Feb 2;21:58. doi: 10.1186/s13020-026-01335-x (PMC12866375; doi:10.1186/s13020-026-01335-x)
Supplement: Supplementary file 1 — Additional file 1. [file 13020_2026_1335_MOESM1_ESM.docx]

**Table S1 Table of secondary differential metabolites in psoralidin**

| **Compounds** | **VIP** | ***P* value** | **Fold Change** | **Type** |
| --- | --- | --- | --- | --- |
| Tomatidine galactoside | 1.8753 | 0.0000 | 4.6150 | up |
| cis-3-Hexen-1-ol | 1.5754 | 0.0076 | 1.4450 | up |
| Triton X-100 | 1.7699 | 0.0066 | 1.2432 | up |
| Butanoic acid, 4-((1,2-dioxohexadecyl)amino)-, ethyl ester | 1.7316 | 0.0087 | 1.2059 | up |
| 1,3,7,8-Tetramethylxanthine | 1.9365 | 0.0035 | 2.0608 | up |
| Riboflavin | 1.9804 | 0.0037 | 1.3695 | up |
| Chlorthalidone | 1.8148 | 0.0106 | 0.4592 | down |
| Chalcomoracin | 1.7466 | 0.0174 | 0.7156 | down |
| Sphinganine | 1.8205 | 0.0053 | 1.2590 | up |
| LPC(O-16:0/2:0) | 1.9067 | 0.0053 | 1.2962 | up |
| Pro-Lys | 2.1095 | 0.0026 | 2.8030 | up |
| Spermine | 2.1059 | 0.0303 | 8.1088 | up |
| Iodotyrosine | 1.6118 | 0.0221 | 0.6847 | down |
| D-Glutamic acid | 2.0961 | 0.0092 | 0.5388 | down |
| Peonidin-3-glucoside | 1.8603 | 0.0044 | 0.5451 | down |
| Deltaline | 1.6201 | 0.0211 | 0.7574 | down |
| Cyanate | 1.9649 | 0.0047 | 1.2996 | up |
| Cytidine | 2.4158 | 0.0000 | 0.7351 | down |
| Salicylamide | 2.3015 | 0.0001 | 0.5263 | down |
| Oxprenolol | 2.4836 | 0.0000 | 0.4814 | down |
| Phenacemide | 1.9393 | 0.0298 | 2.4519 | up |
| Arecoline | 1.9632 | 0.0009 | 0.7633 | down |
| Methionine | 1.5592 | 0.0155 | 0.7287 | down |
| 3-Carboxypropyltrimethylammonium | 1.6909 | 0.0232 | 1.5951 | up |
| Cholesteryl nervonate | 1.5050 | 0.0345 | 2.7906 | up |
| alpha-Hydroxyisobutyric acid | 1.7637 | 0.0143 | 0.7681 | down |
| N-Methylactinodaphnine | 1.6321 | 0.0311 | 0.8084 | down |
| 1-Phenyl-1,3-eicosanedione | 2.0956 | 0.0039 | 0.4559 | down |
| Tetracosanal | 1.5592 | 0.0169 | 1.2275 | up |
| Cosmosiin | 1.9226 | 0.0462 | 34.1250 | up |
| Aclarubicin | 1.6691 | 0.0265 | 0.6464 | down |
| L-Glutamic acid | 2.0175 | 0.0065 | 0.5842 | down |
| Glutamine | 1.6710 | 0.0086 | 1.4579 | up |
| Phytosphingosine | 1.7136 | 0.0087 | 1.2080 | up |
| (5alpha,14alpha)-14-Methylcholestan-3-one | 1.9464 | 0.0071 | 1.2393 | up |
| Isoniazid | 1.7603 | 0.0137 | 1.5715 | up |
| M-Cymene | 1.5422 | 0.0271 | 0.6587 | down |
| Laricitrin | 1.9258 | 0.0015 | 0.7154 | down |
| Estrone | 1.6194 | 0.0444 | 0.5642 | down |
| 2,5-Dihydroxypyridine | 2.1089 | 0.0051 | 4.4178 | up |
| Epinephrine | 1.8934 | 0.0016 | 0.7348 | down |
| L-Name | 1.5159 | 0.0234 | 0.6690 | down |
| Hexanoyl-coenzyme a | 1.5820 | 0.0284 | 2.6586 | up |
| Mangostenol | 1.6149 | 0.0320 | 0.7747 | down |
| Maslinic acid | 1.5091 | 0.0251 | 1.2305 | up |
| Fenbendazole | 2.0805 | 0.0006 | 0.7532 | down |
| L-gamma-glutamyl-D-alanine | 1.6108 | 0.0129 | 0.7994 | down |
| 1-Amino-3,3-diethoxypropane | 2.0219 | 0.0183 | 3.0490 | up |
| Carnitine | 1.5403 | 0.0087 | 1.2492 | up |
| Asclepin | 2.4737 | 0.0000 | 3.9778 | up |
| 3'-Dephosphocoenzyme A | 1.6510 | 0.0020 | 1.7937 | up |
| Phosphocholine | 1.7033 | 0.0194 | 1.5351 | up |
| S-(Hydroxymethyl)glutathione | 2.1946 | 0.0057 | 7.3973 | up |
| Docosanamide | 1.7933 | 0.0339 | 0.5935 | down |
| Zidovudine | 1.8002 | 0.0082 | 0.6213 | down |
| Harmaline | 2.5839 | 0.0000 | 8.9322 | up |
| Rucaparib | 1.7355 | 0.0065 | 1.3046 | up |
| N-(2-Phenylethyl)indomethacin Amide | 2.5446 | 0.0000 | 35.1062 | up |
| Se-methylselenocysteine | 1.6851 | 0.0125 | 1.3041 | up |
| Methylarsonite | 1.8129 | 0.0087 | 1.2148 | up |
| Trazodone hydrochloride | 2.3719 | 0.0001 | 1.2214 | up |
| S-Lactoylglutathione | 1.7498 | 0.0119 | 0.3802 | down |
| Geranylgeraniol | 1.5388 | 0.0200 | 1.4574 | up |
| o-Tyrosine | 2.0958 | 0.0000 | 3.2407 | up |
| Bardoxolone methyl | 1.9200 | 0.0197 | 1.6304 | up |
| 4-(4-Hydroxyaniline)-6,7-Dimethoxyquinazoline-JANEX-1 | 1.5663 | 0.0251 | 1.2715 | up |
| 2-Phenylethanol | 1.7176 | 0.0073 | 0.7965 | down |
| LPC(18:0/0:0) | 1.9170 | 0.0054 | 1.2756 | up |
| 4-O-beta-D-mannopyranosyl-N-acetyl-D-glucosamine | 1.9143 | 0.0109 | 3.4458 | up |
| Urobilin | 1.7394 | 0.0081 | 0.3375 | down |
| Indole-3-lactic acid | 2.1105 | 0.0006 | 1.2830 | up |
| Ethyl arachidonate | 1.6894 | 0.0058 | 0.7851 | down |
| Tapentadol | 2.4148 | 0.0002 | 0.3829 | down |
| Tyrosyl-Valine | 1.9972 | 0.0006 | 2.2229 | up |
| Allopurinol riboside | 1.5770 | 0.0199 | 1.2215 | up |
| Edulitine | 1.7830 | 0.0071 | 1.4736 | up |
| Flavin adenine dinucleotide | 2.0803 | 0.0004 | 1.2556 | up |
| Succinic acid-2,2,3,3-d4 | 2.0330 | 0.0009 | 2.9860 | up |
| Mesterolone | 1.6170 | 0.0274 | 1.6944 | up |
| Neomycin | 1.6898 | 0.0073 | 1.3644 | up |
| Spinosyn D | 1.9923 | 0.0031 | 1.8394 | up |
| Dinex | 1.6915 | 0.0141 | 0.7765 | down |
| Flavin Single Nucleotide(FMN) | 1.9258 | 0.0068 | 1.5036 | up |
| Prephenic acid | 1.6317 | 0.0234 | 1.2344 | up |
| Ipconazole | 1.9348 | 0.0357 | 2.7038 | up |
| Spermidine | 2.0897 | 0.0120 | 2.5554 | up |
| Tuaminoheptane | 2.1889 | 0.0006 | 0.6303 | down |
| LPC(20:3/0:0) | 1.7787 | 0.0033 | 1.3852 | up |
| 2-Aminobenzoic acid | 1.8235 | 0.0054 | 0.7631 | down |
| Enantio-PAF C-16 | 2.3595 | 0.0001 | 1.3866 | up |
| Behenic acid | 2.2429 | 0.0010 | 0.5517 | down |
| 13-Docosenamide | 1.8124 | 0.0405 | 0.5506 | down |
| phosphoribosyl-ATP | 2.1314 | 0.0011 | 1.7340 | up |
| Ethyl oleate | 2.0191 | 0.0411 | 0.3475 | down |
| Diethanolamine | 1.9910 | 0.0029 | 0.7367 | down |
| Valeric acid | 1.5701 | 0.0232 | 0.6557 | down |
| n-Methyl-l-valyl-l-tryptophanol | 2.3719 | 0.0000 | 12.0241 | up |
| magnesium;methyl (3R,11Z,12R,21S,22S)-16-acetyl-11-ethylidene-12,17,21,26-tetramethyl-4-oxo-22-[3-oxo-3-[(2E,6E,10E)-3,7,11,15-tetramethylhexadeca-2,6,10,14-tetraenoxy]propyl]-23,25-diaza-7,24-diazanidahexacyclo[18.2.1.15,8.110,13.115,18.02,6]hexacosa-1,5,8(26),9,13(25),14,16,18,20(23)-nonaene-3-carboxylate | 1.5541 | 0.0486 | 2.6002 | up |
| alpha-Ionone | 2.3970 | 0.0000 | 1.2114 | up |
| Val-Thr-Phe-Glu-Ser | 1.7462 | 0.0327 | 2.0320 | up |
| Val-Thr-Leu-Asp-Met | 1.5468 | 0.0150 | 0.6903 | down |
| Val-Ile-Leu-Asp | 1.6248 | 0.0014 | 1.6605 | up |
| Val-Glu-Phe-Asp | 2.0359 | 0.0000 | 5.7820 | up |
| Tyr-Ser-Thr | 2.0281 | 0.0022 | 1.5629 | up |
| Tyr-Glu-Cys | 1.9721 | 0.0058 | 1.5563 | up |
| Tyr-Glu-Asp-Tyr-Val | 2.0230 | 0.0010 | 1.5522 | up |
| Trp-Arg-Met | 1.5535 | 0.0278 | 0.8031 | down |
| TG(10:0/12:0/i-12:0) | 1.5222 | 0.0212 | 1.2629 | up |
| Ser-Ile-Phe-Glu | 2.6616 | 0.0000 | 7.1916 | up |
| S-Glutathionyl-L-cysteine | 1.9115 | 0.0316 | 0.5175 | down |
| Repaglinide aromatic amine | 1.8676 | 0.0039 | 1.2602 | up |
| Pro-Gln-Tyr | 2.3299 | 0.0000 | 5.4764 | up |
| Phe-Ile-Phe-Met-Gly | 1.6939 | 0.0139 | 1.2213 | up |
| Phe-Gln-Arg-Lys | 2.3190 | 0.0000 | 16.4089 | up |
| Phe-Arg-Phe | 2.2310 | 0.0004 | 0.7835 | down |
| Pentadecylamine | 1.8269 | 0.0040 | 1.2262 | up |
| PS(18:2(9Z,12Z)/20:4(5Z,8Z,11Z,14Z)) | 2.0756 | 0.0046 | 1.6464 | up |
| PE-NMe2(15:0/15:0) | 1.7888 | 0.0071 | 2.6681 | up |
| PE-NMe(18:1(11Z)/22:5(4Z,7Z,10Z,13Z,16Z)) | 2.1385 | 0.0040 | 1.6596 | up |
| PE-NMe(18:0/22:5(4Z,7Z,10Z,13Z,16Z)) | 1.7336 | 0.0450 | 0.4304 | down |
| PE-NMe(18:0/18:1(11Z)) | 1.5497 | 0.0481 | 2.9256 | up |
| PE-NMe(15:0/22:5(7Z,10Z,13Z,16Z,19Z)) | 1.5223 | 0.0310 | 4.2818 | up |
| Notoginsenoside H | 2.0625 | 0.0029 | 1.5378 | up |
| Nitrosylsulfuric acid | 1.6013 | 0.0362 | 0.8111 | down |
| N-methylundec-10-enamide | 1.8204 | 0.0023 | 0.6478 | down |
| N-acetyl-N6,O-didemethylpuromycin | 2.6224 | 0.0000 | 84.9088 | up |
| N-(2-(3-(piperazin-1-ylmethyl)imidazo[2,1-b]thiazol-6-yl)phenyl)quinoxaline-2-carboxamide | 1.5312 | 0.0271 | 0.8112 | down |
| Methapyrilene | 1.5535 | 0.0254 | 2.4145 | up |
| Met-Ser-Phe-Thr-Phe | 2.4019 | 0.0000 | 1.3215 | up |
| Met-Glu-Leu-Tyr-Arg | 1.9779 | 0.0033 | 1.2402 | up |
| Lys-Met-Arg | 1.5424 | 0.0256 | 1.2713 | up |
| Leu-Gln-Arg-Arg | 1.5430 | 0.0196 | 1.2921 | up |
| Leu-Cys-Ala | 2.0453 | 0.0003 | 1.7023 | up |
| Leu-Ala-Thr-Lys | 1.5690 | 0.0017 | 1.6573 | up |
| LPC(22:4/0:0) | 1.6122 | 0.0294 | 1.2373 | up |
| LPC(0:0/22:5) | 1.8441 | 0.0058 | 1.2790 | up |
| LPC(0:0/20:2) | 1.5707 | 0.0181 | 1.2845 | up |
| Imipraminoxide | 1.8656 | 0.0178 | 0.6700 | down |
| Ile-Thr-Gln | 1.7527 | 0.0123 | 1.4256 | up |
| Ile-Thr-Ala | 2.0277 | 0.0013 | 1.2587 | up |
| Ile-Arg-Asn-Asp | 1.7672 | 0.0009 | 2.5776 | up |
| His-Tyr-Gln | 2.0005 | 0.0001 | 6.0441 | up |
| His-Phe-Tyr | 2.5072 | 0.0000 | 22.6117 | up |
| His-Phe-Arg-Asp | 1.7817 | 0.0046 | 1.4077 | up |
| His-Ala-Leu-Glu | 1.9308 | 0.0053 | 0.8223 | down |
| Gly-Lys-Lys-Gln-Leu | 1.6247 | 0.0114 | 1.7779 | up |
| Gly-Gly-Asn-Glu-Glu | 2.4250 | 0.0000 | 17.7564 | up |
| Glu-Tyr-Asp-Lys | 2.5725 | 0.0004 | 44.3170 | up |
| DL-alpha-Tocopherol | 2.0522 | 0.0106 | 0.4008 | down |
| DG(18:4(6Z,9Z,12Z,15Z)/20:4(8Z,11Z,14Z,17Z)/0:0) | 1.5875 | 0.0085 | 1.4257 | up |
| Cys-Ala-Pro-Pro-Thr | 1.6818 | 0.0178 | 0.6978 | down |
| Cetaben | 2.2797 | 0.0005 | 0.4479 | down |
| Asn-Val-Asp-Glu-Val | 2.0116 | 0.0017 | 2.0255 | up |
| Arg-Val-Ile-Trp-Gly | 1.7841 | 0.0107 | 1.2457 | up |
| Arg-Thr-Ala-Arg | 1.6601 | 0.0010 | 1.6871 | up |
| Ala-Thr-Tyr-Lys | 1.6097 | 0.0060 | 1.7062 | up |
| Ala-Ile-Ala | 1.5090 | 0.0011 | 1.7309 | up |
| Ala-Asn-Arg-Val-Thr | 1.8163 | 0.0077 | 1.3632 | up |
| 5-Heptenoic acid, 7-((1R,2R,3R,5S)-3,5-dihydroxy-2-((3R)-3-hydroxy-5-phenylpentyl)cyclopentyl)-, 4-(3-thioxo-3H-1,2-dithiol-5-yl)phenyl ester, (5Z)- | 1.5960 | 0.0140 | 1.2245 | up |
| 4,4'-Diapophytofluene | 1.5246 | 0.0294 | 1.2180 | up |
| 3-[3-[(4-Fluorophenyl)sulfonylamino]-1,2,3,4-tetrahydrocarbazol-9-yl]propanoic acid | 1.7170 | 0.0137 | 2.4406 | up |
| 3,6-Dimethylsalicylyl-CoA | 1.5057 | 0.0453 | 3.1254 | up |
| 2-amino-4-({1-[(carboxymethyl)-C-hydroxycarbonimidoyl]-2-[(4-hydroxy-5-oxo-1,7-diphenylheptan-3-yl)sulfanyl]ethyl}-C-hydroxycarbonimidoyl)butanoic acid | 1.7497 | 0.0153 | 2.0112 | up |
| 2-Methylene-4-oxopentanedioic acid | 1.7359 | 0.0064 | 0.7186 | down |
| 2-Hydroxy-2H-benzo[h]chromene-2-carboxylate | 1.5391 | 0.0230 | 0.8110 | down |
| 2-Hexaprenyl-3-methyl-6-methoxy-1,4 benzoquinone | 1.5035 | 0.0153 | 0.6240 | down |
| 1-Octadecyl-glycero-3-phosphate | 1.7472 | 0.0078 | 1.3391 | up |
| 1-O-Hexadecyl-2-O-(2E-butenoyl)-sn-glyceryl-3-phosphocholine | 1.7641 | 0.0084 | 1.3438 | up |
| 1-(5Z,8Z,11Z,14Z-eicosatetraenoyl)-2-(5Z,8Z,11Z,14Z,17Z-eicosapentaenoyl)-sn-glycerol | 1.8984 | 0.0068 | 1.5690 | up |
| Prunasin | 2.2639 | 0.0001 | 0.6917 | down |
| Citicoline | 1.9686 | 0.0021 | 1.7558 | up |
| Pyroglutamic acid | 1.7300 | 0.0213 | 0.8226 | down |
| 3,3',4'-Trihydroxy-beta,beta-caroten-4-one | 1.9568 | 0.0046 | 1.2194 | up |
| 2'-Deoxyuridine 5'-monophosphate | 1.6612 | 0.0165 | 0.6306 | down |
| 2,4-Di-tert-butylphenol | 2.1058 | 0.0021 | 1.4569 | up |
| 2'-Deoxyuridine | 1.7942 | 0.0052 | 0.7700 | down |
| 2,4,5-Trichlorophenol | 2.1490 | 0.0049 | 0.4863 | down |
| 6,16,24-Trihydroxy-3,8,22,27,29-pentamethyl-11,15,19-trioxaoctacyclo[14.14.1.02,14.04,12.05,10.018,26.020,25.027,31]hentriaconta-2(14),3,5,7,9,12,18(26),20,22,24,29-undecaen-17-one | 2.3499 | 0.0001 | 1.8727 | up |
| Enrofloxacin | 1.6446 | 0.0327 | 0.6404 | down |
| Pyrrolidino PAF C-16 | 1.8600 | 0.0040 | 1.5107 | up |
| Sodium pantothenate | 2.0720 | 0.0008 | 0.7354 | down |
| 1-Hexadecyl-2-butyryl-sn-glycero-3-phosphocholine | 1.9155 | 0.0081 | 1.3090 | up |
| 3',4'-Dimethoxy-2'-hydroxychalcone | 1.7447 | 0.0019 | 3.8873 | up |
| 5,6-EET | 1.7644 | 0.0096 | 1.3500 | up |
| Pantothenic acid | 1.7960 | 0.0047 | 0.6258 | down |
| Pyruvaldehyde | 1.5870 | 0.0135 | 0.6897 | down |
| Epigallocatechin-(4beta->8)-catechin | 2.3884 | 0.0027 | 11.4990 | up |
| Dihydroretrofractamide B | 1.5139 | 0.0352 | 1.7502 | up |
| Platelet activating factor | 1.5460 | 0.0364 | 1.2719 | up |
| N-Phenylacetylphenylalanine | 1.5898 | 0.0286 | 1.4625 | up |
| 5'-Fluoro-5'-deoxyadenosine | 1.7928 | 0.0024 | 2.1104 | up |
| 5-Hydroxy-2-methyl-1H-indole-3-carboxylic acid | 2.4608 | 0.0000 | 1.2571 | up |
| 6-trans-12-epi Leukotriene B4 | 1.7326 | 0.0337 | 1.4571 | up |
| Iliren | 2.3477 | 0.0001 | 1.2224 | up |
| Z-Phenylacetaldoxime | 1.6893 | 0.0150 | 0.7869 | down |
| Phenyl dihydrogen phosphate | 1.5482 | 0.0132 | 0.7516 | down |
| Cytarabine hydrochloride | 1.8046 | 0.0061 | 0.7262 | down |
| Mannitol | 1.8117 | 0.0011 | 1.7424 | up |
| Edulone A | 1.6877 | 0.0082 | 2.6080 | up |
| S-Nitroso-N-acetyl-DL-penicillamine | 1.9173 | 0.0019 | 0.8204 | down |
| Xanthohumol | 1.9545 | 0.0024 | 1.2915 | up |
| Lys-Ser | 1.5144 | 0.0019 | 2.3815 | up |
| DL-Dopa | 1.7426 | 0.0101 | 1.2984 | up |
| Cytidine-5'-diphosphate | 2.1868 | 0.0004 | 1.4357 | up |
| 7,8-Dimethyl-10-(2'-acetoxyethyl)isoalloxazine | 2.0675 | 0.0008 | 1.3285 | up |
| Cyclic AMP | 1.7509 | 0.0127 | 0.7975 | down |
| Tenoxicam | 1.7404 | 0.0162 | 1.2758 | up |
| Maltitol | 2.2504 | 0.0001 | 1.4264 | up |
| Eleganin | 1.9754 | 0.0032 | 1.3344 | up |
| Parathion | 1.5138 | 0.0391 | 0.6393 | down |
| Thr-His | 2.2334 | 0.0003 | 1.2148 | up |
| Gangaleoidin | 1.6353 | 0.0134 | 0.7822 | down |
| Thalsimine | 1.5256 | 0.0298 | 1.2263 | up |
| 7-Methylxanthine | 2.0193 | 0.0014 | 0.4841 | down |
| Myosmine | 1.8261 | 0.0044 | 0.7239 | down |
| D-Mannose | 2.0221 | 0.0014 | 1.2574 | up |
| Cellobiono-1,5-lactone | 1.6924 | 0.0258 | 1.2001 | up |
| (+)-Isoxanthochymol | 1.6411 | 0.0206 | 1.7868 | up |
| O-Desmethylnaproxen | 2.0314 | 0.0015 | 1.5131 | up |
| Apigenin | 2.4282 | 0.0000 | 1.8122 | up |
| Cycleanine | 1.6482 | 0.0371 | 1.4834 | up |
| 1-Methyladenine | 2.4507 | 0.0000 | 1.2551 | up |
| Diosgenin | 2.0083 | 0.0022 | 1.2597 | up |
| Lipomycin | 2.0538 | 0.0202 | 5.7284 | up |
| Estradiol | 1.6882 | 0.0202 | 0.6820 | down |
| Glucotropaeolin | 1.7848 | 0.0090 | 0.7847 | down |
| Pantetheine | 1.6730 | 0.0030 | 2.4339 | up |
| Baicalein | 1.6159 | 0.0244 | 0.6532 | down |
| Ononin | 2.1468 | 0.0020 | 1.6483 | up |
| Daphnin | 2.0821 | 0.0026 | 1.2124 | up |
| Adenosine-5'-phosphosulfate | 1.6821 | 0.0198 | 1.5186 | up |
| Santonin | 1.8713 | 0.0069 | 0.7994 | down |
| Artemetin | 2.1561 | 0.0064 | 6.4803 | up |
| Dyphylline | 1.8557 | 0.0025 | 2.7129 | up |
| 2-Methyl-3-hydroxybutyric acid | 1.9488 | 0.0107 | 1.5611 | up |
| Codeinone | 1.8647 | 0.0008 | 5.8968 | up |
| Obeticholic acid | 1.6664 | 0.0154 | 0.7153 | down |
| Homoeriodictyol | 2.2445 | 0.0002 | 1.2375 | up |
| alpha-CEHC | 1.9359 | 0.0079 | 1.7342 | up |
| Citrulline | 2.4914 | 0.0000 | 1.2530 | up |
| UDP-xylose | 1.9945 | 0.0038 | 0.6067 | down |
| 2-(6-(4-Aminophenoxy)-3-oxo-3H-xanthen-9-yl)benzoic acid | 2.4362 | 0.0000 | 1.2275 | up |
| 1,2-Ethanediol, 1-(3-methoxy-4-(sulfooxy)phenyl)- | 1.8779 | 0.0026 | 0.6906 | down |
| 2'-Deoxyinosine 5'-monophosphate | 2.4100 | 0.0000 | 0.7953 | down |
| Hippomane factor M1 | 1.5434 | 0.0476 | 5.8926 | up |
| 4-Nitrocatechol | 1.9328 | 0.0037 | 1.3002 | up |
| 3-Methylcrotonylglycine | 1.9604 | 0.0068 | 1.6238 | up |
| Chlorambucil | 1.6507 | 0.0142 | 1.4808 | up |
| Tretinoin | 1.6831 | 0.0292 | 0.5541 | down |
| Galactose-uridine-5'-diphosphate | 1.9433 | 0.0368 | 0.3354 | down |
| Ureidoisobutyric Acid | 1.6156 | 0.0125 | 1.4587 | up |
| Geniposidic acid | 1.7665 | 0.0129 | 0.7301 | down |
| Suxibuzone | 1.9286 | 0.0000 | 6.0717 | up |
| D-Fructose 6-Phosphate-Disodium Salt | 1.6815 | 0.0063 | 0.7002 | down |
| Vidarabine | 1.6340 | 0.0289 | 1.2965 | up |
| Asp-Tyr | 1.6355 | 0.0160 | 1.2432 | up |
| Lumiracoxib | 2.1733 | 0.0003 | 0.7467 | down |
| L-Alanine, N-L-asparaginyl- | 1.9757 | 0.0037 | 0.7738 | down |
| (3b,16a,21b,22a)-12-Oleanene-3,16,21,23,28-pentol-22-angeloyloxy-23-al | 1.9455 | 0.0013 | 1.7957 | up |
| 5-Acetylamino-6-amino-3-methyluracil | 1.5788 | 0.0212 | 1.2925 | up |
| Cysteinyldopa | 1.6764 | 0.0211 | 0.6560 | down |
| Tetragastrin | 1.6052 | 0.0307 | 1.2657 | up |
| 8,9-DiHETrE | 1.5472 | 0.0375 | 1.2466 | up |
| Loganin | 1.9176 | 0.0059 | 1.2741 | up |
| Madecassic acid | 1.5194 | 0.0437 | 7.3872 | up |
| 24,25-Diacetylvulgaroside | 2.1826 | 0.0017 | 1.4859 | up |
| Pantethine | 2.4935 | 0.0000 | 0.6114 | down |
| Bexarotene | 1.9346 | 0.0006 | 1.3457 | up |
| 3'-Sulfogalactosylceramide | 2.1886 | 0.0004 | 1.4755 | up |
| 4,5alpha-Dihydrocortisone | 1.8458 | 0.0039 | 1.3873 | up |
| 2-Indolecarboxylic acid | 2.1772 | 0.0001 | 1.3247 | up |
| (20S)-20-Hydroxypregn-4-en-3-one | 1.7419 | 0.0313 | 1.4672 | up |
| Pseudouridine | 1.9251 | 0.0016 | 0.7701 | down |
| 3,4-Dihydroxybenzaldehyde | 2.1186 | 0.0030 | 0.6036 | down |
| Phosphoenolpyruvate | 1.9632 | 0.0183 | 0.5755 | down |
| Calcium pantothenate | 1.6948 | 0.0092 | 0.6749 | down |
| N-[2-(3,4-Dihydroxyphenyl)ethyl]hexadecanamide | 2.1606 | 0.0008 | 0.7752 | down |
| Hydroflumethiazide | 1.6720 | 0.0365 | 0.6856 | down |
| H-Trp-ser-OH | 2.1869 | 0.0011 | 1.6542 | up |
| Uridine-5'-diphosphate-glucose | 1.8502 | 0.0286 | 0.3717 | down |
| Dihydroxyfumaric acid | 1.8281 | 0.0222 | 20.2862 | up |
| D-Arabinose-5-phosphate | 1.6933 | 0.0143 | 1.3549 | up |
| 17-phenyl-trinor-PGF2alpha isopropyl ester | 1.7733 | 0.0248 | 0.7263 | down |
| 2'-Adenylic acid | 1.6044 | 0.0273 | 0.7041 | down |
| Arvanil | 1.7934 | 0.0105 | 1.4533 | up |
| 3-[1-(3-Aminopropyl)-1H-indol-3-YL]-4-(1-methyl-1H-indol-3-YL)-1H-pyrrole-2,5-dione | 2.4785 | 0.0002 | 2.2814 | up |
| Eudesobovatol A | 1.8275 | 0.0106 | 5.3972 | up |
| Succinimide | 1.9650 | 0.0024 | 0.5937 | down |
| Rosiglitazone | 2.0233 | 0.0252 | 3.0050 | up |
| Xanthopterin | 1.5868 | 0.0214 | 0.7844 | down |
| 2-[2-[19-Acetamido-6-(3,4-dicarboxybutanoyloxy)-16,18-dihydroxy-5,9-dimethylicosan-7-yl]oxy-2-oxoethyl]butanedioic acid | 2.4378 | 0.0000 | 1.4562 | up |
| Dalfopristin | 2.1860 | 0.0004 | 1.3165 | up |
| gamma-Glutamylglutamate | 1.8961 | 0.0108 | 0.6076 | down |
| L-Cysteinesulfinic acid | 1.6826 | 0.0461 | 86.8181 | up |
| Mizolastine | 2.0750 | 0.0010 | 1.3250 | up |
| 4-Chlorophenol | 2.1493 | 0.0003 | 0.8063 | down |
| Urdamycin B | 2.0957 | 0.0035 | 0.5632 | down |
| Neoacrimarine E | 1.9438 | 0.0081 | 1.8361 | up |
| bicyclo-PGE2 | 1.5235 | 0.0275 | 0.6713 | down |
| UDP-D-galactose | 1.9648 | 0.0197 | 0.3176 | down |
| Tyr-Thr-Ala-Glu | 1.9192 | 0.0000 | 4.2890 | up |
| Tyr-Glu-Gln-Asp | 2.2075 | 0.0003 | 0.6581 | down |
| Tumonoic acid A | 1.5441 | 0.0356 | 1.2647 | up |
| Trypanothione | 1.7777 | 0.0088 | 0.7123 | down |
| Trp-Nap-OH | 2.3622 | 0.0000 | 1.2592 | up |
| Ser-Gln-Leu-Lys | 1.7745 | 0.0070 | 0.7559 | down |
| Riesling acetal | 1.5935 | 0.0220 | 0.7644 | down |
| Phe-Gln-Ala-Arg | 1.5704 | 0.0209 | 1.4395 | up |
| Phaclofen | 1.7407 | 0.0069 | 1.2448 | up |
| O-Demethylpuromycin | 1.8728 | 0.0092 | 1.2084 | up |
| Neu5Ac2-6Gal1-4GlcNAcSp | 2.0928 | 0.0010 | 0.6811 | down |
| N-[2-(4-Oxo-1-phenyl-1,3,8-triazaspiro[4.5]decan-8-yl)ethyl]naphthalene-2-carboxamide | 2.3091 | 0.0003 | 9.9178 | up |
| N-Desmethyl tapentadol | 2.0473 | 0.0043 | 1.5726 | up |
| Lupinate | 1.8966 | 0.0018 | 1.5643 | up |
| Luminespib | 2.2158 | 0.0011 | 1.4058 | up |
| LPS(22:6/0:0) | 1.5207 | 0.0161 | 1.5404 | up |
| LPI(16:0/0:0) | 1.7446 | 0.0070 | 1.2339 | up |
| L-a-Lysophosphatidylserine | 2.1664 | 0.0007 | 1.4142 | up |
| L-Oleandrosyl-oleandolide | 2.1162 | 0.0003 | 1.2620 | up |
| Ile-Ser-Val-Asp | 2.2624 | 0.0001 | 11.3629 | up |
| Ile-Glu-Val-Asp-Leu | 1.6406 | 0.0100 | 3.5614 | up |
| Ile-Asn-Val-Asp | 2.1502 | 0.0008 | 0.6169 | down |
| His-Glu-Phe-Glu | 1.6959 | 0.0201 | 1.3448 | up |
| Halofenozide | 1.7242 | 0.0093 | 1.2135 | up |
| Gomisin E | 2.5070 | 0.0001 | 25.6624 | up |
| Glu-Gln-Phe-Arg | 1.9912 | 0.0005 | 0.6032 | down |
| Ganolucidic Acid E | 1.6917 | 0.0252 | 2.8505 | up |
| Deacetoxy(7)-7-oxokhivorinic acid | 1.6549 | 0.0063 | 0.7441 | down |
| DG(20:4(5Z,8Z,11Z,14Z)/18:1(11Z)/0:0) | 2.0736 | 0.0004 | 1.6838 | up |
| D-ribofuranose 5-phosphate | 2.0124 | 0.0197 | 3.5479 | up |
| D-Gal alpha 1->6D-Gal alpha 1->6D-Glucose | 1.9594 | 0.0002 | 4.1120 | up |
| Clopyralid | 2.0651 | 0.0020 | 0.7854 | down |
| Chlorophyllide a | 2.3987 | 0.0003 | 9.3612 | up |
| Chivosazole F | 1.5534 | 0.0090 | 1.8706 | up |
| Capsicum annuum Fluorescent chlorophyll catabolite | 1.5907 | 0.0152 | 0.8150 | down |
| CDP-N-methylethanolamine | 1.6918 | 0.0333 | 0.6492 | down |
| Buprenorphine Glucuronide | 1.7760 | 0.0070 | 1.3781 | up |
| Barbital | 2.3762 | 0.0002 | 1.3217 | up |
| Avermectin B2a aglycone | 2.0222 | 0.0033 | 1.5081 | up |
| Aspartylmethionine | 2.1617 | 0.0002 | 0.7302 | down |
| Aspartylasparagine | 1.5756 | 0.0305 | 0.5012 | down |
| Asn-Thr-Asp-Arg | 2.2443 | 0.0000 | 13.7902 | up |
| Asn-Arg-Gln-Lys | 1.8672 | 0.0120 | 4.1343 | up |
| Amebacilin | 2.2817 | 0.0004 | 0.6428 | down |
| 8-Acetoxypinoresinol 4-glucoside | 2.3416 | 0.0000 | 0.6005 | down |
| 6,8a-Seco-6,8a-deoxy-5-oxoavermectin''1a'' aglycone | 2.0888 | 0.0005 | 1.9292 | up |
| 4S,5S-antillatoxin A | 1.6103 | 0.0204 | 3.8224 | up |
| 4-hydroxy Nonenal Glutathione-d3 | 1.8436 | 0.0075 | 0.8036 | down |
| 4-hydroxy Nonenal Glutathione | 1.5457 | 0.0384 | 1.6659 | up |
| 4-Thia-1-azabicyclo[3.2.0]heptane-2-carboxylic acid, 6-[[(2R)-2-amino-2-(4-hydroxyphenyl)acetyl]amino]-3,3-dimethyl-7-oxo-, (2S,5R,6R)- | 1.5822 | 0.0390 | 1.5833 | up |
| 4-(Butylamino)benzoic acid | 1.9583 | 0.0035 | 1.3881 | up |
| 4'-Hydroxyanigorootin | 1.6218 | 0.0220 | 1.4087 | up |
| 3-deoxy-D-arabino-heptulosonate-7-phosphate | 2.1549 | 0.0000 | 0.3687 | down |
| 3,4,6-trihydroxy-5-oxo-1-(3,5,7-trihydroxy-3,4-dihydro-2H-chromen-2-yl)benzo[7]annulene-8-carboxylic acid | 1.9908 | 0.0045 | 0.7631 | down |
| 3,4,5-trihydroxy-6-({5-hydroxy-8,8-dimethyl-4-oxo-2-phenyl-4H,8H-pyrano[2,3-f]chromen-3-yl}oxy)oxane-2-carboxylic acid | 1.5788 | 0.0038 | 3.2353 | up |
| 3,4,5-trihydroxy-6-({3-hydroxy-8,8-dimethyl-4-oxo-2-phenyl-4H,8H-pyrano[2,3-f]chromen-5-yl}oxy)oxane-2-carboxylic acid | 1.5679 | 0.0007 | 3.0152 | up |
| 2-dehydro-3-deoxy-D-glucaric acid | 1.5573 | 0.0348 | 0.5164 | down |
| 2-amino-4-({1-[(carboxymethyl)-C-hydroxycarbonimidoyl]-2-{[2-hydroxy-3-(4-hydroxy-2-methoxyphenyl)-1-phenylpropyl]sulfanyl}ethyl}-C-hydroxycarbonimidoyl)butanoic acid | 2.3157 | 0.0002 | 1.2152 | up |
| 2-amino-4-({1-[(carboxymethyl)-C-hydroxycarbonimidoyl]-2-{[2-hydroxy-1-(7-methoxy-2-oxo-2H-chromen-6-yl)-3-oxobutyl]sulfanyl}ethyl}-C-hydroxycarbonimidoyl)butanoic acid | 2.2191 | 0.0004 | 1.2004 | up |
| 2-[(4-{2-[(4-Cyclohexylbutyl)(cyclohexylcarbamoyl)amino]ethyl}phenyl)sulfanyl]-2-methylpropanoic acid | 1.5002 | 0.0204 | 2.6665 | up |
| 2,2':5',2''-Terthiophene | 1.6734 | 0.0103 | 0.7416 | down |
| 15-Demethylaclacinomycin T | 1.6410 | 0.0079 | 0.5493 | down |
| 1-Erucoylglycerol-3-phosphate | 2.2386 | 0.0003 | 1.6606 | up |
| 1-Caffeoyl-beta-D-glucose | 1.7424 | 0.0055 | 1.9252 | up |
| 1-(2-hydroxy-5-(trifluoromethyl)phenyl)-5-(trifluoromethyl)-1H-benzo[d]imidazol-2(3H)-one | 1.5119 | 0.0251 | 0.4442 | down |
| (3b,5a,25R)-3-Hydroxyspirostan-6-one 3-[2-acetylarabinosyl-(1->6)-glucoside] | 2.3101 | 0.0001 | 1.2836 | up |
| (3R,6R)-1,3,4,6-tetrachlorocyclohexa-1,4-diene | 1.7606 | 0.0130 | 0.5491 | down |
| (2R,6S,7R,9R,12S,16S)-6-hydroxy-15-[(1S)-1-[5-(hydroxymethyl)-4-methyl-6-oxo-2,3-dihydropyran-2-yl]ethyl]-2,16-dimethyl-8-oxapentacyclo[9.7.0.02,7.07,9.012,16]octadec-4-en-3-one | 2.5333 | 0.0000 | 1.2768 | up |
| (2R)-2-amino-3-hydrosulfonylpropanoic acid | 1.5789 | 0.0487 | 63.5230 | up |
