# Supplementary material for: Psoralidin, a main compound in Psoraleae Fructus, induces hepatotoxicity by impeding lipid oxidative catabolism and aggravating lipid accumulation in mice
Source: Chin Med. 2026 Feb 2;21:58. doi: 10.1186/s13020-026-01335-x (PMC12866375; doi:10.1186/s13020-026-01335-x)
Supplement: Supplementary file 2 — Additional file 2. [file 13020_2026_1335_MOESM2_ESM.docx]

**Table S2** **Informations of differential proteins in psoralidin**

| **Accession** | **Gene** | **Fold Change** | ***P* value** | **Regulated type** |
| --- | --- | --- | --- | --- |
| A0A1W2P872 | Nova2 | 7.2271978 | 0.0000076 | up |
| A2AJI0 | Map7d1 | 6.1000973 | 0.0000866 | up |
| A2AKK5 | Acnat1 | 38.3456064 | 0.0000012 | up |
| A2APY7 | Ndufaf5 | 8.5224547 | 0.0000020 | up |
| A2ARZ3 | Fsip2 | 6.3312252 | 0.0000149 | up |
| A2BH40 | Arid1a | 0.1815388 | 0.0018763 | down |
| A2RSJ4 | Bltp3b | 0.1553079 | 0.0013833 | down |
| A3KGB4 | Tbc1d8b | 6.5301361 | 0.0000027 | up |
| A6H8H2 | Dennd4c | 0.0951325 | 0.0007010 | down |
| B7ZMP1 | Xpnpep3 | 13.4208424 | 0.0000001 | up |
| B8JK39 | Itga9 | 6.5110365 | 0.0000053 | up |
| B9EJ86 | Osbpl8 | 10.6608510 | 0.0000000 | up |
| E9PYK3 | Parp4 | 6.1625171 | 0.0000053 | up |
| E9Q394 | Akap13 | 9.0021057 | 0.0004595 | up |
| E9Q8D0 | Dnajc21 | 22.6169873 | 0.0005994 | up |
| O35075 | Vps26c | 14.8787307 | 0.0000004 | up |
| O35099 | Map3k5 | 17.8963116 | 0.0000105 | up |
| O35445 | Rnf5 | 0.1649224 | 0.0046221 | down |
| O35623 | Bet1 | 0.1323046 | 0.0000257 | down |
| O35643 | Ap1b1 | 9.1869567 | 0.0000200 | up |
| O35683 | Ndufa1 | 5.4148724 | 0.0003647 | up |
| O35943 | Fxn | 9.1797120 | 0.0000090 | up |
| O54804 | Chka | 8.0194387 | 0.0003259 | up |
| O54824 | Il16 | 5.3898454 | 0.0008524 | up |
| O54983 | Crym | 6.9750122 | 0.0016900 | up |
| O55042 | Snca | 0.1412713 | 0.0000933 | down |
| O55102 | Bloc1s1 | 0.1883469 | 0.0000023 | down |
| O70228 | Atp9a | 21.8713524 | 0.0002276 | up |
| O70481 | Ubr1 | 0.1715896 | 0.0000143 | down |
| O70503 | Hsd17b12 | 5.1262765 | 0.0000163 | up |
| O70579 | Slc25a17 | 11.5983611 | 0.0000085 | up |
| O88597 | Becn1 | 23.7637594 | 0.0000269 | up |
| O88668 | Creg1 | 13.9587158 | 0.0004248 | up |
| O88792 | F11r | 0.1787158 | 0.0000447 | down |
| O88796 | Rpp30 | 8.1881999 | 0.0000006 | up |
| O88833 | Cyp4a10 | 5.0582021 | 0.0000411 | up |
| O88947 | F10 | 0.1763358 | 0.0001221 | down |
| O88952 | Lin7c | 12.6069072 | 0.0001282 | up |
| O88983 | Stx8 | 6.3328634 | 0.0000041 | up |
| O89086 | Rbm3 | 8.7464596 | 0.0012648 | up |
| O89106 | Fhit | 5.9521464 | 0.0000824 | up |
| P00158 | Mt-Cyb | 76.4007176 | 0.0000003 | up |
| P00397 | Mtco1 | 14.6968564 | 0.0000465 | up |
| P00687 | Amy1 | 0.1307124 | 0.0000120 | down |
| P01800 | -- | 7.1417751 | 0.0000051 | up |
| P01869 | Ighg1 | 8.1383028 | 0.0025610 | up |
| P01901 | H2-K1 | 0.0748584 | 0.0069758 | down |
| P02798 | Mt2 | 22.4741880 | 0.0212194 | up |
| P03893 | mt-Nd2 | 0.1626028 | 0.0000415 | down |
| P03921 | Mtnd5 | 15.2000367 | 0.0003699 | up |
| P06346 | H2-Ab1 | 9.5850565 | 0.0000222 | up |
| P06537 | Nr3c1 | 0.1694475 | 0.0000041 | down |
| P07214 | Sparc | 14.2034199 | 0.0000018 | up |
| P08103 | Hck | 39.1857580 | 0.0000000 | up |
| P08680 | Alas2 | 43.5548737 | 0.0000802 | up |
| P08730 | Krt13 | 6.5334923 | 0.0003557 | up |
| P10493 | Nid1 | 10.5180643 | 0.0000002 | up |
| P11588 | Mup1 | 156.5978269 | 0.0009904 | up |
| P11589 | Mup2 | 5.1140575 | 0.0291516 | up |
| P11928 | Oas1a | 0.1891287 | 0.0000001 | down |
| P12790 | Cyp2b9 | 0.0564875 | 0.0000152 | down |
| P13516 | Scd1 | 0.1562233 | 0.0399037 | down |
| P13597 | Icam1 | 6.6635608 | 0.0001056 | up |
| P14069 | S100a6 | 5.9351089 | 0.0052348 | up |
| P16330 | Cnp | 0.1242982 | 0.0227127 | down |
| P18826 | Phka1 | 6.8023297 | 0.0000055 | up |
| P21300 | Akr1b7 | 5.6762430 | 0.0001217 | up |
| P21570 | Ang | 14.2885904 | 0.0000288 | up |
| P22366 | Myd88 | 12.2116280 | 0.0000004 | up |
| P23780 | Glb1 | 0.1555871 | 0.0000581 | down |
| P27005 | S100a8 | 6.8555085 | 0.0011933 | up |
| P27600 | Gna12 | 13.3596788 | 0.0000330 | up |
| P28654 | Dcn | 0.1555977 | 0.0003546 | down |
| P28867 | Prkcd | 6.5632529 | 0.0085554 | up |
| P29533 | Vcam1 | 7.9819629 | 0.0000890 | up |
| P31996 | Cd68 | 5.4184423 | 0.0012486 | up |
| P35283 | Rab12 | 7.1720545 | 0.0000236 | up |
| P35822 | Ptprk | 0.1826777 | 0.0000065 | down |
| P35922 | Fmr1 | 54.7758178 | 0.0000006 | up |
| P40240 | Cd9 | 5.4765708 | 0.0003553 | up |
| P42128 | Foxk1 | 5.5778461 | 0.0000115 | up |
| P42227 | Stat3 | 5.1140472 | 0.0000038 | up |
| P42703 | Lifr | 6.1233474 | 0.0000017 | up |
| P46425 | Gstp2 | 12.0892474 | 0.0000948 | up |
| P48437 | Prox1 | 6.6048920 | 0.0000470 | up |
| P49290 | Epx | 0.1115886 | 0.0000018 | down |
| P50428 | Arsa | 6.9316620 | 0.0005391 | up |
| P51163 | Uros | 10.2053671 | 0.0000262 | up |
| P52795 | Efnb1 | 5.0282811 | 0.0005003 | up |
| P53569 | Cebpz | 5.8348000 | 0.0002131 | up |
| P53612 | Rabggtb | 0.1394424 | 0.0000204 | down |
| P54310 | Lipe | 25.4421356 | 0.0000008 | up |
| P55012 | Slc12a2 | 6.7296892 | 0.0000047 | up |
| P55098 | Pex2 | 5.6636620 | 0.0015089 | up |
| P56376 | Acyp1 | 17.3088955 | 0.0000768 | up |
| P56394 | Cox17 | 0.1543823 | 0.0001299 | down |
| P56655 | Cyp2c38 | 5.5192971 | 0.0001934 | up |
| P58064 | Mrps6 | 6.6142581 | 0.0000003 | up |
| P58404 | Strn4 | 0.1210550 | 0.0001184 | down |
| P58802 | Tbc1d10a | 5.1135264 | 0.0003409 | up |
| P59326 | Ythdf1 | 16.1769725 | 0.0000067 | up |
| P60521 | Gabarapl2 | 5.1537838 | 0.0002130 | up |
| P61211 | Arl1 | 7.1676392 | 0.0021760 | up |
| P61971 | Nutf2 | 11.8068526 | 0.0000002 | up |
| P62254 | Ube2g1 | 19.2476587 | 0.0000588 | up |
| P62274 | Rps29 | 5.9822870 | 0.0001104 | up |
| P62305 | Snrpe | 9.3130642 | 0.0000894 | up |
| P62313 | Lsm6 | 0.1574659 | 0.0000178 | down |
| P62320 | Snrpd3 | 5.8656246 | 0.0000023 | up |
| P62488 | Polr2g | 0.1891430 | 0.0000134 | down |
| P62715 | Ppp2cb | 0.0421804 | 0.0002558 | down |
| P62748 | Hpcal1 | 5.4702253 | 0.0000247 | up |
| P62878 | Rbx1 | 0.1504402 | 0.0000070 | down |
| P62996 | Tra2b | 13.4875726 | 0.0000172 | up |
| P63001 | Rac1 | 0.1720248 | 0.0007631 | down |
| P63082 | Atp6v0c | 5.1861560 | 0.0001287 | up |
| P63168 | Dynll1 | 5.7829173 | 0.0000082 | up |
| P63213 | Gng2 | 0.1952324 | 0.0002787 | down |
| P63300 | Selenow | 11.5421208 | 0.0000746 | up |
| P68368 | Tuba4a | 0.1703707 | 0.0000229 | down |
| P68372 | Tubb4b | 0.1477182 | 0.0000016 | down |
| P70266 | Pfkfb1 | 5.7481215 | 0.0000010 | up |
| P70268 | Pkn1 | 0.1364999 | 0.0000663 | down |
| P70362 | Ufd1 | 5.9909149 | 0.0000421 | up |
| P70429 | Evl | 0.1782590 | 0.0006385 | down |
| P70691 | Ugt1a2 | 0.0403861 | 0.0002002 | down |
| P82343 | Renbp | 7.3554034 | 0.0012486 | up |
| P83877 | Txnl4a | 10.9873172 | 0.0000010 | up |
| P83917 | Cbx1 | 8.2853957 | 0.0000102 | up |
| P97287 | Mcl1 | 5.5404791 | 0.0042220 | up |
| P97379 | G3bp2 | 0.1961378 | 0.0000038 | down |
| P97470 | Ppp4c | 5.5233073 | 0.0000179 | up |
| P97471 | Smad4 | 0.1598030 | 0.0000394 | down |
| P97493 | Txn2 | 40.8188944 | 0.0002653 | up |
| P97770 | Thumpd3 | 0.0571949 | 0.0007157 | down |
| P97789 | Xrn1 | 0.1615668 | 0.0001223 | down |
| P97819 | Pla2g6 | 5.8004956 | 0.0001138 | up |
| P97868 | Rbbp6 | 0.1726269 | 0.0011783 | down |
| P99024 | Tubb5 | 16.6608147 | 0.0001413 | up |
| Q00897 | Serpina1d | 5.6386302 | 0.0000593 | up |
| Q00899 | Yy1 | 0.0888061 | 0.0000001 | down |
| Q05117 | Acp5 | 7.9202045 | 0.0006136 | up |
| Q08024 | Cbfb | 5.2259144 | 0.0002119 | up |
| Q0VGB7 | Ppp4r2 | 7.8738998 | 0.0000201 | up |
| Q14CH7 | Aars2 | 14.0523423 | 0.0000246 | up |
| Q2L4X1 | Bzw2 | 0.0664548 | 0.0000070 | down |
| Q3TLS3 | Gdpgp1 | 31.3140563 | 0.0000021 | up |
| Q3TPE9 | Ankmy2 | 8.9199758 | 0.0005063 | up |
| Q3TRM4 | Pnpla6 | 14.0885746 | 0.0003596 | up |
| Q3TWW8 | Srsf6 | 5.0827700 | 0.0000032 | up |
| Q3U5Q7 | Cmpk2 | 0.0682126 | 0.0000082 | down |
| Q3U7R1 | Esyt1 | 8.2390024 | 0.0001702 | up |
| Q3UCV8 | Otulin | 6.3241686 | 0.0000003 | up |
| Q3UD01 | Atxn7l3b | 0.0499834 | 0.0000194 | down |
| Q3UE37 | Ube2z | 5.0248220 | 0.0001227 | up |
| Q3UHN9 | Ndst1 | 5.9344083 | 0.0000060 | up |
| Q3UKC1 | Tax1bp1 | 18.3141810 | 0.0015604 | up |
| Q3UMT1 | Ppp1r12c | 0.1076003 | 0.0000494 | down |
| Q3UNA4 | Nxt2 | 5.2896142 | 0.0000205 | up |
| Q3UQ28 | Pxdn | 13.5537289 | 0.0000042 | up |
| Q4KML4 | Abracl | 5.9717057 | 0.0000109 | up |
| Q505B7 | Zbtb8os | 5.3156715 | 0.0000034 | up |
| Q5F285 | Tmem256 | 20.0993763 | 0.0000002 | up |
| Q5F2E8 | Taok1 | 5.6902703 | 0.0002318 | up |
| Q5FW60 | Mup20 | 8.9919872 | 0.0053692 | up |
| Q5SFM8 | Rbm27 | 0.0830496 | 0.0000996 | down |
| Q5SNZ0 | Ccdc88a | 9.7170102 | 0.0002332 | up |
| Q5SSH8 | Cyb5d2 | 5.2601151 | 0.0000284 | up |
| Q5SV85 | Synrg | 0.0291360 | 0.0001122 | down |
| Q5U419 | Mfsd3 | 6.2584105 | 0.0000042 | up |
| Q5U5M8 | Bloc1s3 | 9.0018115 | 0.0004629 | up |
| Q60680 | Chuk | 6.0050829 | 0.0000989 | up |
| Q60870 | Reep5 | 0.1410389 | 0.0001807 | down |
| Q60991 | Cyp7b1 | 40.6609147 | 0.0000014 | up |
| Q61103 | Dpf2 | 11.3586386 | 0.0002261 | up |
| Q61107 | Gbp4 | 8.8127068 | 0.0003952 | up |
| Q61285 | Abcd2 | 0.0142827 | 0.0000001 | down |
| Q61411 | Hras | 0.0838443 | 0.0001800 | down |
| Q61420 | Slc35a1 | 17.5803529 | 0.0001541 | up |
| Q61462 | Cyba | 5.2371698 | 0.0007408 | up |
| Q61493 | Rev3l | 0.0464970 | 0.0000580 | down |
| Q61581 | Igfbp7 | 11.0466372 | 0.0015952 | up |
| Q61585 | G0s2 | 8.4435393 | 0.0000491 | up |
| Q61753 | Phgdh | 0.1264063 | 0.0004756 | down |
| Q62193 | Rpa2 | 10.4050815 | 0.0000016 | up |
| Q62392 | Phlda1 | 11.0339716 | 0.0002008 | up |
| Q62393 | Tpd52 | 18.4123567 | 0.0000009 | up |
| Q62422 | Ostf1 | 5.5620127 | 0.0003040 | up |
| Q62433 | Ndrg1 | 0.1167306 | 0.0000021 | down |
| Q62441 | Tle4 | 0.1034895 | 0.0001512 | down |
| Q63810 | Ppp3r1 | 0.1214067 | 0.0000018 | down |
| Q63829 | Commd3 | 9.7354901 | 0.0000058 | up |
| Q63836 | Selenbp2 | 10.5376018 | 0.0011171 | up |
| Q63850 | Nup62 | 0.1640741 | 0.0000176 | down |
| Q63961 | Eng | 0.1904521 | 0.0002585 | down |
| Q64191 | Aga | 9.5486406 | 0.0000141 | up |
| Q64282 | Ifit1 | 0.1108826 | 0.0026312 | down |
| Q64310 | Surf4 | 5.4749913 | 0.0000021 | up |
| Q64327 | Mea1 | 0.0874485 | 0.0020204 | down |
| Q64373 | Bcl2l1 | 6.7906487 | 0.0000072 | up |
| Q64435 | Ugt1a6 | 5.3330880 | 0.0000357 | up |
| Q64505 | Cyp7a1 | 6.4956555 | 0.0000103 | up |
| Q64525 | Hist2h2bb | 24.3112397 | 0.0000079 | up |
| Q64669 | Nqo1 | 0.1322068 | 0.0002007 | down |
| Q66GT5 | Ptpmt1 | 0.1939849 | 0.0004235 | down |
| Q66JZ4 | TCAIM | 11.2247528 | 0.0000021 | up |
| Q68FL4 | Ahcyl2 | 31.6627246 | 0.0000194 | up |
| Q69ZN7 | Myof | 66.4013655 | 0.0003947 | up |
| Q69ZP3 | Pnkd | 0.1993918 | 0.0000007 | down |
| Q6NSR8 | Npepl1 | 7.2422760 | 0.0000781 | up |
| Q6NXN1 | Szrd1 | 6.6332204 | 0.0000065 | up |
| Q6P1F6 | Ppp2r2a | 12.5743988 | 0.0000001 | up |
| Q6P8I4 | Pcnp | 0.1947881 | 0.0000003 | down |
| Q6P9R1 | Ddx51 | 7.8921137 | 0.0000002 | up |
| Q6PAK3 | Prmt8 | 18.0149451 | 0.0000030 | up |
| Q6PCP5 | Mff | 9.5843591 | 0.0000002 | up |
| Q6PD10 | Ip6k1 | 5.0618621 | 0.0004346 | up |
| Q6PHQ8 | Naa35 | 6.4894558 | 0.0000017 | up |
| Q6PJN8 | Dalrd3 | 54.5724134 | 0.0002084 | up |
| Q6RHR9 | Magi1 | 8.1688801 | 0.0001400 | up |
| Q6ZPZ3 | Zc3h4 | 0.1679370 | 0.0014164 | down |
| Q6ZQ88 | Kdm1a | 0.0435591 | 0.0000354 | down |
| Q6ZQL4 | Wdr43 | 5.3632155 | 0.0000514 | up |
| Q6ZWU9 | Rps27 | 11.9810709 | 0.0000062 | up |
| Q6ZWY3 | Rps27l | 0.0746090 | 0.0008064 | down |
| Q78HU7 | Gypc | 0.0870579 | 0.0004641 | down |
| Q7TMC8 | Fcsk | 5.2884545 | 0.0000810 | up |
| Q7TMQ7 | Wdr91 | 6.1635008 | 0.0001810 | up |
| Q7TMY4 | Thoc7 | 0.1099074 | 0.0000004 | down |
| Q7TPH6 | Mycbp2 | 18.5927551 | 0.0002348 | up |
| Q7TQE6 | Maco1 | 7.2244579 | 0.0000035 | up |
| Q7TQK1 | Ints7 | 7.7863445 | 0.0000390 | up |
| Q7TSI3 | Ppp6r1 | 0.1725189 | 0.0027542 | down |
| Q7TT37 | Elp1 | 6.1704871 | 0.0000081 | up |
| Q80SW1 | Ahcyl1 | 0.1969401 | 0.0002236 | down |
| Q80TN4 | Dnajc16 | 0.1136889 | 0.0004562 | down |
| Q80TP3 | Ubr5 | 0.0925994 | 0.0001429 | down |
| Q80TV8 | Clasp1 | 5.2477646 | 0.0000128 | up |
| Q80U95 | Ube3c | 5.5336711 | 0.0000037 | up |
| Q80UP8 | Slc20a2 | 29.3820544 | 0.0000687 | up |
| Q80UU1 | Ankzf1 | 0.1822215 | 0.0011469 | down |
| Q80V94 | Ap4e1 | 0.1045834 | 0.0000113 | down |
| Q80W00 | Ppp1r10 | 12.4499112 | 0.0000453 | up |
| Q80WB5 | Ntaq1 | 5.4919805 | 0.0005230 | up |
| Q80XC3 | Usp6nl | 10.4692560 | 0.0011795 | up |
| Q80XD1 | Chn2 | 0.1263261 | 0.0002640 | down |
| Q80XK6 | Atg2b | 0.1504350 | 0.0000103 | down |
| Q80ZI6 | Lrsam1 | 0.0909164 | 0.0001490 | down |
| Q80ZW2 | Them6 | 7.8580752 | 0.0000005 | up |
| Q810D6 | Grwd1 | 8.1743928 | 0.0000019 | up |
| Q8BFQ4 | Wdr82 | 0.1438244 | 0.0000091 | down |
| Q8BGB2 | Ttc7a | 6.0135827 | 0.0006318 | up |
| Q8BGB5 | Limd2 | 8.6357468 | 0.0003553 | up |
| Q8BGB7 | Enoph1 | 11.2827798 | 0.0001380 | up |
| Q8BGG9 | Acnat2 | 0.0893101 | 0.0019120 | down |
| Q8BGQ1 | Vipas39 | 38.9431925 | 0.0000027 | up |
| Q8BGS2 | Bola2 | 0.1900512 | 0.0001218 | down |
| Q8BGV8 | Mief1 | 6.6768261 | 0.0000337 | up |
| Q8BGW1 | Fto | 14.0781083 | 0.0000016 | up |
| Q8BGZ4 | Cdc23 | 0.1776757 | 0.0009632 | down |
| Q8BH51 | Cox14 | 14.7742122 | 0.0000040 | up |
| Q8BH64 | Ehd2 | 6.1509883 | 0.0000062 | up |
| Q8BHB4 | Wdr3 | 0.1486235 | 0.0000128 | down |
| Q8BHC4 | Dcakd | 6.3437714 | 0.0001406 | up |
| Q8BHG3 | Tmem30b | 0.1353901 | 0.0007197 | down |
| Q8BHL5 | Elmo2 | 24.6379090 | 0.0000192 | up |
| Q8BJ05 | Zc3h14 | 0.1216921 | 0.0004121 | down |
| Q8BJ48 | Nagpa | 8.7082289 | 0.0000819 | up |
| Q8BKF1 | Polrmt | 53.7198158 | 0.0000657 | up |
| Q8BL74 | Gtf3c2 | 82.8789034 | 0.0001599 | up |
| Q8BM88 | Ctso | 6.6756404 | 0.0000503 | up |
| Q8BMQ2 | Gtf3c4 | 5.3250105 | 0.0002046 | up |
| Q8BRV5 | Kiaa1671 | 6.5780739 | 0.0000558 | up |
| Q8BS40 | Cptp | 26.3677536 | 0.0000203 | up |
| Q8BTI9 | Pik3cb | 5.4262182 | 0.0000042 | up |
| Q8BU85 | Msrb3 | 8.9717600 | 0.0000001 | up |
| Q8BUY9 | Pggt1b | 0.1653281 | 0.0000013 | down |
| Q8BVZ1 | Plin5 | 7.6323565 | 0.0003026 | up |
| Q8BW94 | Dnah3 | 0.1680850 | 0.0007658 | down |
| Q8BWG8 | Arrb1 | 5.2127766 | 0.0000789 | up |
| Q8BWG9 | Orai1 | 7.5166633 | 0.0000374 | up |
| Q8BWJ3 | Phka2 | 6.9226280 | 0.0000234 | up |
| Q8BWN8 | Acot4 | 10.3263835 | 0.0000000 | up |
| Q8BWR2 | Pithd1 | 22.2570632 | 0.0000004 | up |
| Q8BXL7 | Arfrp1 | 9.1920669 | 0.0000013 | up |
| Q8BZA9 | Tigar | 8.1336371 | 0.0000105 | up |
| Q8C052 | Map1s | 9.3532205 | 0.0000011 | up |
| Q8C4X7 | Minar2 | 17.8656877 | 0.0000053 | up |
| Q8C547 | Heatr5b | 17.1965899 | 0.0000000 | up |
| Q8C7V3 | Utp15 | 0.0603462 | 0.0000414 | down |
| Q8C7V8 | Ccdc134 | 7.1937819 | 0.0000046 | up |
| Q8CAK3 | Shfl | 0.1883301 | 0.0000186 | down |
| Q8CB27 | Yod1 | 6.5115095 | 0.0001462 | up |
| Q8CBE3 | Wdr37 | 268.0389046 | 0.0002233 | up |
| Q8CBY8 | Dctn4 | 6.4274342 | 0.0000009 | up |
| Q8CCB4 | Vps53 | 6.4504998 | 0.0015434 | up |
| Q8CCK0 | Macroh2a2 | 9.4944208 | 0.0000023 | up |
| Q8CEI1 | Bola3 | 6.5535270 | 0.0000018 | up |
| Q8CG16 | C1ra | 15.5325517 | 0.0000007 | up |
| Q8CH40 | Nudt6 | 8.1005796 | 0.0000021 | up |
| Q8CHC4 | Synj1 | 9.2233459 | 0.0000002 | up |
| Q8CI95 | Osbpl11 | 5.8223092 | 0.0010099 | up |
| Q8CIN4 | Pak2 | 5.6533964 | 0.0001446 | up |
| Q8CIV8 | Tbce | 10.8148660 | 0.0000009 | up |
| Q8CJ53 | Trip10 | 7.4475903 | 0.0000038 | up |
| Q8CJ96 | Rassf8 | 0.1026321 | 0.0000142 | down |
| Q8JZY2 | Commd10 | 10.5685232 | 0.0000365 | up |
| Q8K0L9 | Zbtb20 | 5.0640100 | 0.0000000 | up |
| Q8K1A5 | Tmem41b | 0.0962773 | 0.0000025 | down |
| Q8K1E6 | Alkbh3 | 0.1388572 | 0.0000019 | down |
| Q8K1L5 | Ppp1r11 | 12.8867621 | 0.0002987 | up |
| Q8K1X1 | Wdr11 | 7.0235593 | 0.0000037 | up |
| Q8K211 | Slc31a1 | 0.1175836 | 0.0000623 | down |
| Q8K245 | Uvrag | 7.0398094 | 0.0024805 | up |
| Q8K2A7 | Ints10 | 5.6009712 | 0.0000241 | up |
| Q8K2F0 | Brd3 | 11.9638818 | 0.0000651 | up |
| Q8K2I4 | Manba | 31.6648512 | 0.0000007 | up |
| Q8K2K6 | Agfg1 | 0.1648506 | 0.0000056 | down |
| Q8K2Q0 | Commd9 | 6.2595091 | 0.0000442 | up |
| Q8K354 | Cbr3 | 0.0252142 | 0.0000784 | down |
| Q8K358 | Pigu | 17.0359363 | 0.0000801 | up |
| Q8K3G5 | Vrk3 | 6.3126224 | 0.0000175 | up |
| Q8K3G9 | Appl2 | 0.1148333 | 0.0000001 | down |
| Q8K3W0 | Babam2 | 10.1079519 | 0.0000298 | up |
| Q8K409 | Polb | 5.7679051 | 0.0000994 | up |
| Q8K4M5 | Commd1 | 6.2784897 | 0.0000403 | up |
| Q8K5B2 | Mcfd2 | 0.1597062 | 0.0008026 | down |
| Q8R0G7 | Spns1 | 15.0436017 | 0.0000008 | up |
| Q8R121 | Serpina10 | 0.1874116 | 0.0000185 | down |
| Q8R1A4 | Dock7 | 78.5120565 | 0.0000333 | up |
| Q8R1K1 | Ubac2 | 10.6506186 | 0.0000645 | up |
| Q8R1S4 | Mtss1 | 30.3271754 | 0.0000063 | up |
| Q8R1S9 | Slc38a4 | 7.9899001 | 0.0011934 | up |
| Q8R1T4 | Slc35a3 | 7.0772791 | 0.0000453 | up |
| Q8R2M2 | Dnttip2 | 0.1921776 | 0.0007286 | down |
| Q8R2Q8 | Bst2 | 0.0567855 | 0.0002226 | down |
| Q8R3P0 | Aspa | 5.6196057 | 0.0010561 | up |
| Q8R3R8 | Gabarapl1 | 6.3186649 | 0.0000922 | up |
| Q8R4R6 | Nup35 | 6.6348746 | 0.0000097 | up |
| Q8R5A6 | Tbc1d22a | 6.9336854 | 0.0000017 | up |
| Q8R5F7 | Ifih1 | 0.1957801 | 0.0007095 | down |
| Q8VC42 | Rmc1 | 5.1638260 | 0.0000016 | up |
| Q8VC52 | Rbpms2 | 7.8018790 | 0.0015466 | up |
| Q8VCI0 | Plbd1 | 26.6157277 | 0.0000161 | up |
| Q8VCY6 | Utp6 | 0.0594964 | 0.0022628 | down |
| Q8VD00 | Tmem97 | 12.2375951 | 0.0000635 | up |
| Q8VD66 | Abhd4 | 24.2758946 | 0.0000216 | up |
| Q8VD75 | Hip1 | 16.0751011 | 0.0004387 | up |
| Q8VE80 | Thoc3 | 11.4074767 | 0.0000041 | up |
| Q8VE92 | Rbm4b | 0.0676109 | 0.0000078 | down |
| Q8VE97 | Srsf4 | 0.1283538 | 0.0000553 | down |
| Q8VEH3 | Arl8a | 0.1754861 | 0.0000781 | down |
| Q8VEH6 | Zng1 | 5.1565371 | 0.0010313 | up |
| Q8VHI3 | Pofut2 | 5.3736015 | 0.0000301 | up |
| Q8VHK1 | Caskin2 | 15.4680903 | 0.0000014 | up |
| Q8VHL5 | Crygn | 6.7394192 | 0.0005868 | up |
| Q8VI94 | Oasl1 | 0.1950524 | 0.0002437 | down |
| Q91V16 | Etfrf1 | 10.1827502 | 0.0000402 | up |
| Q91VC7 | Ppp1r14a | 5.3849570 | 0.0000440 | up |
| Q91VE6 | Nifk | 12.0033396 | 0.0001165 | up |
| Q91VR7 | Map1lc3a | 6.5452778 | 0.0001213 | up |
| Q91VU0 | Fam3c | 7.1370906 | 0.0000141 | up |
| Q91VU7 | Pus7 | 20.4404276 | 0.0000060 | up |
| Q91VW3 | Sh3bgrl3 | 5.9882670 | 0.0000078 | up |
| Q91W39 | Ncoa5 | 6.1766276 | 0.0001149 | up |
| Q91WC9 | Daglb | 12.4611172 | 0.0000024 | up |
| Q91WG2 | Rabep2 | 5.6877678 | 0.0000349 | up |
| Q91WI7 | Itfg2 | 8.4862289 | 0.0009810 | up |
| Q91WR3 | Ascc2 | 0.0769584 | 0.0007982 | down |
| Q91WZ8 | Dtnbp1 | 19.4983800 | 0.0000107 | up |
| Q91XV3 | Basp1 | 0.1522314 | 0.0009788 | down |
| Q91YL3 | Uckl1 | 10.7488361 | 0.0000001 | up |
| Q91ZI0 | Celsr3 | 5.5458127 | 0.0001449 | up |
| Q91ZW2 | Pofut1 | 43.2799258 | 0.0000278 | up |
| Q920R0 | Als2 | 5.3516388 | 0.0005449 | up |
| Q922H1 | Prmt3 | 0.1420280 | 0.0004748 | down |
| Q922S4 | Pde2a | 5.1521994 | 0.0049192 | up |
| Q923D4 | Sf3b5 | 6.1315396 | 0.0001193 | up |
| Q99J56 | Derl1 | 5.4001180 | 0.0007407 | up |
| Q99J93 | Ifitm2 | 7.1059229 | 0.0004393 | up |
| Q99JF5 | Mvd | 27.1661870 | 0.0000000 | up |
| Q99JH1 | Rpp25l | 0.1120722 | 0.0000007 | down |
| Q99JH8 | Kdelr1 | 5.0504704 | 0.0000134 | up |
| Q99JI6 | Rap1b | 5.0643754 | 0.0009118 | up |
| Q99JX4 | Eif3m | 5.4474389 | 0.0000015 | up |
| Q99L27 | Gmpr2 | 7.1548867 | 0.0000053 | up |
| Q99L28 | Rsl24d1 | 5.9649487 | 0.0000726 | up |
| Q99L43 | Cds2 | 29.5780657 | 0.0000006 | up |
| Q99L48 | Nmd3 | 5.0976248 | 0.0001203 | up |
| Q99LG2 | Tnpo2 | 6.9412204 | 0.0003409 | up |
| Q99MU3 | Adar | 0.1971879 | 0.0000739 | down |
| Q99N93 | Mrpl16 | 13.2442330 | 0.0000004 | up |
| Q99NB8 | Ubqln4 | 0.0916097 | 0.0000215 | down |
| Q99P27 | Pla2g12b | 5.1346180 | 0.0000032 | up |
| Q99P31 | Hspbp1 | 11.7474378 | 0.0000157 | up |
| Q99P72 | Rtn4 | 0.1895327 | 0.0000038 | down |
| Q99PI5 | Lpin2 | 0.1331301 | 0.0001091 | down |
| Q9CPW9 | Metap1d | 6.0235571 | 0.0000041 | up |
| Q9CQ43 | Dut | 8.0603998 | 0.0000120 | up |
| Q9CQ91 | Ndufa3 | 5.5259819 | 0.0000048 | up |
| Q9CQA1 | Trappc5 | 5.8823563 | 0.0000008 | up |
| Q9CQA9 | Ntpcr | 7.4767400 | 0.0000398 | up |
| Q9CQB2 | Mcrip2 | 27.5411428 | 0.0000011 | up |
| Q9CQC8 | Spg21 | 7.5431587 | 0.0005356 | up |
| Q9CQE3 | Mrps17 | 5.3393019 | 0.0012452 | up |
| Q9CQL6 | Mrpl35 | 5.1789803 | 0.0000005 | up |
| Q9CQS8 | Sec61b | 0.1924570 | 0.0000094 | down |
| Q9CQW3 | Proz | 0.1265497 | 0.0003142 | down |
| Q9CQW9 | Ifitm3 | 0.0731220 | 0.0000087 | down |
| Q9CQY6 | Uqcc2 | 0.0959705 | 0.0003634 | down |
| Q9CQZ1 | Hsbp1 | 20.4679984 | 0.0000026 | up |
| Q9CR10 | Oxld1 | 6.6980817 | 0.0000374 | up |
| Q9CR41 | Hypk | 0.1776103 | 0.0125639 | down |
| Q9CR76 | Tmem186 | 5.3693146 | 0.0003448 | up |
| Q9CR89 | Ergic2 | 0.0401765 | 0.0000511 | down |
| Q9CRA7 | Dmac2l | 0.1772593 | 0.0000195 | down |
| Q9CSU0 | Rprd1b | 12.4055044 | 0.0000008 | up |
| Q9CW46 | Raver1 | 0.0738888 | 0.0003097 | down |
| Q9CWE0 | Mtfr1l | 6.0593145 | 0.0041554 | up |
| Q9CWH5 | Trmt11 | 11.2974199 | 0.0005449 | up |
| Q9CWQ0 | Dph5 | 11.2338772 | 0.0000570 | up |
| Q9CX98 | Cyp2u1 | 6.6615285 | 0.0014765 | up |
| Q9CXV1 | Sdhd | 17.6034590 | 0.0000109 | up |
| Q9CYK1 | Wars2 | 0.1220055 | 0.0002005 | down |
| Q9CYL5 | Glipr2 | 11.1843715 | 0.0000104 | up |
| Q9CYV5 | Tmem135 | 5.1898510 | 0.0002180 | up |
| Q9CZ69 | Cmtm6 | 9.0925980 | 0.0016367 | up |
| Q9CZB0 | Sdhc | 10.3717076 | 0.0002688 | up |
| Q9CZG3 | Commd8 | 13.9291917 | 0.0000025 | up |
| Q9CZG9 | Pdzd11 | 0.0655047 | 0.0000532 | down |
| Q9CZY3 | Ube2v1 | 0.0300013 | 0.0007345 | down |
| Q9D0B0 | Srsf9 | 6.1080170 | 0.0004780 | up |
| Q9D0J4 | Arl2 | 0.1214039 | 0.0000996 | down |
| Q9D0L4 | Adck1 | 0.0339361 | 0.0005551 | down |
| Q9D0L7 | Armc10 | 6.0374682 | 0.0078822 | up |
| Q9D0Q7 | Mrpl45 | 0.1544230 | 0.0000000 | down |
| Q9D0R9 | Wdr89 | 10.0729606 | 0.0001159 | up |
| Q9D0Z3 | Tmem53 | 8.9378467 | 0.0000014 | up |
| Q9D136 | Ogfod3 | 15.0577180 | 0.0000927 | up |
| Q9D154 | Serpinb1a | 0.1685390 | 0.0000773 | down |
| Q9D1F4 | Akt1s1 | 0.1569286 | 0.0000228 | down |
| Q9D1H7 | Get4 | 22.3063560 | 0.0005144 | up |
| Q9D1I5 | Mcee | 5.0187240 | 0.0000015 | up |
| Q9D289 | Trappc6b | 0.1759676 | 0.0000023 | down |
| Q9D2M8 | Ube2v2 | 19.5424420 | 0.0000129 | up |
| Q9D2R6 | Coa3 | 13.0899573 | 0.0001626 | up |
| Q9D3D9 | Atp5f1d | 0.1222688 | 0.0005424 | down |
| Q9D7E4 | -- | 0.1608823 | 0.0016369 | down |
| Q9D7M1 | Gid8 | 6.9671610 | 0.0001837 | up |
| Q9D7X3 | Dusp3 | 6.4234064 | 0.0001734 | up |
| Q9D892 | Itpa | 6.8178803 | 0.0000004 | up |
| Q9D975 | Srxn1 | 0.1970564 | 0.0170974 | down |
| Q9D9K3 | Aven | 5.3425010 | 0.0001366 | up |
| Q9DB96 | Ngdn | 9.9142184 | 0.0000021 | up |
| Q9DBA6 | Tysnd1 | 0.1419738 | 0.0001797 | down |
| Q9DBM0 | Abcg8 | 8.1451297 | 0.0000007 | up |
| Q9DBQ7 | Scyl3 | 6.9250510 | 0.0001401 | up |
| Q9DBR3 | Armc8 | 9.7921731 | 0.0000002 | up |
| Q9DBX2 | Pdcl | 16.8471027 | 0.0000419 | up |
| Q9DBY8 | Nvl | 0.1812595 | 0.0000263 | down |
| Q9DC48 | Cdc40 | 5.3694500 | 0.0000350 | up |
| Q9DCA5 | Brix1 | 0.1679144 | 0.0006573 | down |
| Q9DCI9 | Mrpl32 | 5.8618845 | 0.0000013 | up |
| Q9DCJ1 | Mlst8 | 0.1356243 | 0.0000203 | down |
| Q9DCL2 | Ciao2a | 16.7232770 | 0.0003786 | up |
| Q9DCV7 | Krt7 | 23.8676432 | 0.0000055 | up |
| Q9EPS3 | Glce | 5.5722436 | 0.0001491 | up |
| Q9EPV8 | Ubl5 | 0.1449764 | 0.0002259 | down |
| Q9EQ21 | Hamp | 5.4685393 | 0.0008035 | up |
| Q9EQZ6 | Rapgef4 | 6.0072697 | 0.0000030 | up |
| Q9ER00 | Stx12 | 5.5742583 | 0.0001668 | up |
| Q9ERL7 | Gmfg | 5.3418459 | 0.0001818 | up |
| Q9ERR7 | Selenof | 35.7236825 | 0.0000009 | up |
| Q9ES46 | Parvb | 0.0798702 | 0.0000016 | down |
| Q9ESJ0 | Xpo4 | 5.1769538 | 0.0004932 | up |
| Q9JHK4 | Rabggta | 7.3270018 | 0.0000003 | up |
| Q9JI46 | Nudt3 | 6.7436472 | 0.0000255 | up |
| Q9JI60 | Lrat | 12.7960267 | 0.0000190 | up |
| Q9JI90 | Rnf14 | 5.1519060 | 0.0000024 | up |
| Q9JII5 | Dazap1 | 14.8783263 | 0.0000232 | up |
| Q9JIS3 | Smco4 | 24.2825236 | 0.0000005 | up |
| Q9JJA2 | Cog8 | 10.9483745 | 0.0000020 | up |
| Q9JJF9 | Sppl2a | 8.1625935 | 0.0000221 | up |
| Q9JJQ0 | Pigb | 9.4795273 | 0.0000030 | up |
| Q9JJW0 | Pxmp4 | 5.7982691 | 0.0001065 | up |
| Q9JJW6 | Alyref2 | 12.1512981 | 0.0000006 | up |
| Q9JJX6 | P2rx4 | 0.1635888 | 0.0000089 | down |
| Q9JKV5 | Scamp4 | 7.1655029 | 0.0000089 | up |
| Q9JLQ2 | Git2 | 9.0533520 | 0.0001807 | up |
| Q9JLZ3 | Auh | 7.0531404 | 0.0002164 | up |
| Q9JMA7 | Cyp3a41b | 0.1889177 | 0.0125686 | down |
| Q9JME7 | Trappc2l | 0.1413083 | 0.0001891 | down |
| Q9JMG7 | Hdgfl3 | 8.6951114 | 0.0001860 | up |
| Q9QXD8 | Limd1 | 5.5288670 | 0.0006028 | up |
| Q9QXK7 | Cpsf3 | 8.3524907 | 0.0000001 | up |
| Q9QY06 | Myo9b | 15.8875877 | 0.0000140 | up |
| Q9QY61 | Irx4 | 9.1852458 | 0.0002554 | up |
| Q9QZC8 | Abhd1 | 6.2503626 | 0.0003555 | up |
| Q9QZR0 | Rnf25 | 5.2944451 | 0.0000028 | up |
| Q9R008 | Mvk | 6.9017094 | 0.0000563 | up |
| Q9R078 | Prkab1 | 8.8975759 | 0.0023408 | up |
| Q9WTN0 | Ggps1 | 8.6259318 | 0.0000012 | up |
| Q9WTQ8 | Timm23 | 8.2481898 | 0.0000216 | up |
| Q9WVC8 | Slc26a3 | 53.3948271 | 0.0181501 | up |
| Q9WVM3 | Anapc7 | 9.8650762 | 0.0000087 | up |
| Q9WVS7 | Map2k5 | 0.1640975 | 0.0029101 | down |
| Q9Z0N1 | Eif2s3x | 8.2953313 | 0.0001921 | up |
| Q9Z0N2 | Eif2s3y | 0.0246598 | 0.0000004 | down |
| Q9Z0P5 | Twf2 | 0.1922619 | 0.0022523 | down |
| Q9Z0S9 | Rabac1 | 0.1214929 | 0.0000003 | down |
| Q9Z211 | Pex11a | 5.1518413 | 0.0000243 | up |
| Q9Z224 | Mocs2 | 0.1812266 | 0.0000632 | down |
| Q9Z266 | Snapin | 5.6573154 | 0.0000721 | up |
| Q9Z2C5 | Mtm1 | 0.1265606 | 0.0045871 | down |
| Q9Z2L6 | Minpp1 | 0.1136252 | 0.0000045 | down |
| Q9Z2V5 | Hdac6 | 16.3124941 | 0.0000102 | up |
